# Genome-Wide Methylation and Gene Expression Changes in Newborn Rats following Maternal Protein Restriction and Reversal by Folic Acid

**DOI:** 10.1371/journal.pone.0082989

**Published:** 2013-12-31

**Authors:** Gioia Altobelli, Irina G. Bogdarina, Elia Stupka, Adrian J. L. Clark, Simon Langley-Evans

**Affiliations:** 1 Centre for Endocrinology, William Harvey Research Centre, Barts and the London School of Medicine and Dentistry, Queen Mary University of London, London, United Kingdom; 2 Institute of Cell and Molecular Bioscience, Barts and the London School of Medicine and Dentistry, Queen Mary University of London, London, United Kingdom; 3 School of Biosciences, University of Nottingham, Sutton Bonington, Loughborough, United Kingdom; RWTH Aachen University Medical School, Germany

## Abstract

A large body of evidence from human and animal studies demonstrates that the maternal diet during pregnancy can programme physiological and metabolic functions in the developing fetus, effectively determining susceptibility to later disease. The mechanistic basis of such programming is unclear but may involve resetting of epigenetic marks and fetal gene expression. The aim of this study was to evaluate genome-wide DNA methylation and gene expression in the livers of newborn rats exposed to maternal protein restriction. On day one postnatally, there were 618 differentially expressed genes and 1183 differentially methylated regions (FDR 5%). The functional analysis of differentially expressed genes indicated a significant effect on DNA repair/cycle/maintenance functions and of lipid, amino acid metabolism and circadian functions. Enrichment for known biological functions was found to be associated with differentially methylated regions. Moreover, these epigenetically altered regions overlapped genetic loci associated with metabolic and cardiovascular diseases. Both expression changes and DNA methylation changes were largely reversed by supplementing the protein restricted diet with folic acid. Although the epigenetic and gene expression signatures appeared to underpin largely different biological processes, the gene expression profile of DNA methyl transferases was altered, providing a potential link between the two molecular signatures. The data showed that maternal protein restriction is associated with widespread differential gene expression and DNA methylation across the genome, and that folic acid is able to reset both molecular signatures.

## Introduction

Non-communicable diseases of adulthood related to obesity (type 2-diabetes, cardiovascular disease, cancer) represent major global public health challenges. Cardiovascular disease in particular is a significant cause of morbidity and mortality [1]. It is widely considered that the primary determinants of risk of non-communicable diseases derive from a combination of environmental factors and the cumulative effects of multiple genetic variations [2]. However, even with increasingly massive studies and detection of ever rarer genetic variants, complete aetiological explanations are elusive [3]. A potential explanation for part of this ‘aetiology gap’ is the phenomenon of fetal programming [4]–[6]. Based originally on the observation that birth weight associated with the prevalence of cardiometabolic disease [7], significant support for its relevance has been provided by the re-creation of this phenomenon in a wide range of animal models [6].

Whilst models of programming of physiological and metabolic function are well-characterized, the underlying mechanisms involved remain unresolved [8]. A basic requirement of fetal programming is that an event *in utero* such as a physical, psychological, metabolic or pharmacological stress results in a change in the fetus that persists through life. This potentially leads to adverse consequences in adulthood [6]. Intrauterine life is characterized by an extensive and complex developmental programme, which involves cell division, growth and differentiation. Differentiation of cells requires stable marking of the genome such that cell types commit to specific lineages [9]. This genome marking process includes modification of DNA by methylation at CpG sites and modification of the chromatin around which DNA is wound. Both of these changes are major determinants of the level of expression of related genes [10]. Consequently, any interference with this process of epigenetic modification is likely to influence patterns of gene expression in a stable manner. This may, in turn, have adverse consequences for the function of the developed organism, either due to prolonged up- or down-regulation of gene expression, or remodeling of tissues during development [6], [11].

The idea that altered DNA methylation following maternal insult is key to this process comes from the observation, in rodent models [12]–[14], that specific genes of interest carry a modified pattern of cytosine methylation that correlates with their level of expression. Generally it is reported that hypomethylation of gene promoters, with up-regulation of expression, occurs in offspring of animals subject to undernutrition during pregnancy. These genes include important modulators of the metabolic phenotype such as *PPAR*α in liver [12], the glucocorticoid receptor in the liver [12] and hippocampus [13], and, in our own studies of a hypertensive rat model, the *At1b* angiotensin receptor in the adrenal glands [14], [15]. Furthermore, the observation that dietary supplementation with single carbon donors, for example folic acid, can reverse the phenotypic changes [16]–[18] in many of these models is consistent with the hypothesis that disturbance of DNA methylation underlies this phenomenon. The expression of DNA methyltransferases may be modified by the maternal diet and this may explain how undernutrition can alter methylation and hence, gene expression [13].

It seems unlikely that a disturbance of DNA methylation in response to maternal undernutrition impacts upon only a few select genes in a few specific tissues. It is more probable that this would be a global phenomenon common across the accessible regions of the genome in most tissues. Sinclair and colleagues reported that brief periconceptual depletion of methyl donors in sheep impacted upon methylation of approximately 4% of the genome in affected lambs [19]. To address these questions we investigated the extent of DNA methylation across the whole genome in the livers of control and programmed animals shortly after birth using the MBD-seq method [20]. Multiple regions of differential methylation were identified and their relationship with differential gene expression was characterized. Given that maternal folic acid supplementation has been shown to reverse programmed changes in DNA methylation, gene expression and functional outcomes [12],[16],[18],[21],[22], we also assessed the impact of supplementation upon whole genome methylation and gene expression.

## Results

### Sample collection

Livers were harvested from male offspring on postnatal day 1 (P1) from rat litters fed on control (18% casein), low protein (9% casein) or low protein with folate- supplemented diets previously described [16], respectively denoted C, MLP and MLP-F. One litter from each group was studied, generating up to 16 offspring in each case. As the aim of our study was to relate methylation and gene expression to programmed phenotypes, we did not include a control with folate supplementation group as there is no evidence that increased folate programmes a distinct postnatal phenotype. Liver was selected for study in view of its size and relative homogeneity and the demonstrated role of several liver-specific genes in insulin resistance. P1 was selected as the sampling time in the expectation that secondary or compensatory gene expression changes would be minimized at this early stage and that DNA methylation profiles would indicate the potential effect of sustained epigenetic programming through the entire pregnancy.

### Gene expression profiles

**Figure 1:**
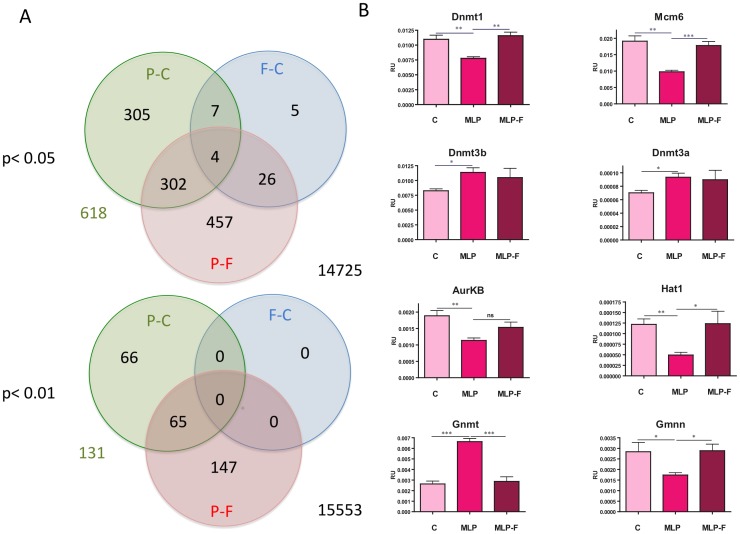
Differentially expressed genes in maternal undernutrition model. (A) Venn's diagrams showing the number of differentially expressed gene probes in comparisons MLP vs. C (P-C), MLP-F vs. C (F-C) and MLP vs. MLP-F (P-F) at FDR 5% (top) and FDR 1% (bottom). Number of gene probes whose expression was unaltered is indicated in black (right). Numbers of altered gene probes in MLP-C contrast at different FDR are indicated in green (left). (B) qRT-PCR mRNA analysis for 8 genes: *Dnmt1*, *Dnmt3a*, *Dnmt3b*, *Aurkb*, *Mcm6*, *Hat1*, *Gnmt* and *Gmnn*. Results are given in relative units, as ratio of number of copies in assessed gene and housekeeping gene GAPDH. ^×^ P<0.05; ^××^ P<0.01; ^×××^ P<0.001. ns P>0.05.

Gene expression in individual livers was assessed using the Illumina RatRef-12 Expression BeadChip (n = 4 RNA samples for each animal group). Differentially expressed genes among the three animal groups were determined according to the procedure outlined in Materials and Methods. Figure 1A shows that at False Discovery Rate (the expected proportion of rejected null hypotheses that are false positives) FDR = 5% 618 gene probes were differentially expressed when control (C) and maternal low protein (MLP) samples were compared. At FDR = 1% 131 genes were still found to be differentially expressed. The complete set of differentially expressed genes in this comparison (FDR 5%) is shown in DATASET S1. Amongst differentially expressed genes there were Glycine N-methyltransferase, *Gnmt*, a key enzyme in 1-carbon metabolism; three key DNA methyltransferases: *Dnmt1* (maintenance), *Dnmt*3a and *Dnmt*3b (*de novo*); components of the *Mcm* gene family; and *polycomb* proteins. Eight of the differentially expressed genes between control and low protein diet samples (including representative genes for *Dnmt* and *Mcm* families) were selected for validation using qPCR. This confirmed the changes seen on arrays in all cases, and also indicated abrogation of these changes in the MLP-F group (Figure 1B). Folate supplementation did not reverse all MLP-induced changes, with *Dnmt3a* and *Dnmt3b* notably irresponsive to the additional folic acid. Functional analysis of the set of differentially expressed genes in MLP vs. C using Gene Set Enrichment Analysis-Molecular Signature Database (GSEA-MSignDB) [23], [24] showed that there was very strong enrichment in DNA repair/cycle/maintenance functions amongst the down-regulated genes; whilst in the up-regulated genes lipid/amino acid metabolism and circadian functions emerged. Moreover, a large proportion of genes in the down-regulated set shared transcription factor binding sites for *E2F* family members in their regulatory regions (within 2 kbp from the TSS). These genes include *enhancer of zeste homolog 2* (*Ezh2*) a histone methyltransferase that is a well-recognised component of the *polycomb* group of proteins as well as an oncogene [25]; and genes from the *Mcm* family. Notably, a large subset of the down-regulated genes overlapped a set of down-regulated genes in an *EZH2* KO experiment [26]. Some up-regulated genes shared transcription factor binding sites for androgen receptor (*AR*) and activating protein 2 (*AP2*). These findings were confirmed by further analysis with GENEGO Software (see Materials and Methods). The complete ontological analysis of these differentially expressed genes can be found in DATASET S2. Previous studies, as well as our unpublished work, have examined hepatic gene expression patterns in this model at later time points suggesting disturbance of more differentiated functions (for example, lipogenesis) at 4, 8 or 12 weeks of age and in older animals [27]–[29]. These changes presumably reflect the evolution of the programmed phenotype with increasing postnatal development as well as secondary consequences of disturbed gene expression.

A greatly reduced number of genes were differentially expressed in the MLP offspring in which the maternal diet had been supplemented with folic acid (MLP-F). 42 genes at FDR 5% (no genes at 1% FDR) were differentially expressed relative to control, implying that folate prevented or reversed the gene expression changes (Fig. 1A). No functional enrichment was found, which is unremarkable given the low number of differentially expressed genes. Assuming that folic acid supplementation may have reversed the MLP phenotype, as expression patterns in MLP-F livers are similar or equal to expression patterns in C livers, we also evaluated differentially expressed genes between MLP and MLP-F. In this comparison we noted a larger number of differentially expressed gene probes (Figure 1A), namely 763, which was similar in size to the previous comparison between MLP-F and C (see DATASET S3). However, only 306 differentially expressed genes (65 at more stringent FDR 1%) were found in both MLP vs. C and MLP vs. MLP-F, indicating potentially more subtle differences between C and MLP-F animals (Figure 1A). The comparison between MLP and MLP-F still retained the differences found in *Mcm*s and *Dnmt*s (except for *Dnmt3a*) genes, indicating that the key pathways that were validated by PCR were indeed altered only in the MLP animals. Further investigation of the genes that were differentially expressed only in the MLP vs. MLP-F comparison indicated enrichment in various metabolic functions (see DATASET S4). The mapped genes (288) that were differentially expressed in both comparisons (MLP vs. C and MLP vs. MLP+F) showed distinctive enrichment in DNA metabolism, catabolism and stress responses (See DATASET S5). In addition to *Dnmts* and *Mcms* genes, we found *polycomb H2afz* (but not *Ezh2*), and complement proteins. Interestingly, a strongly down-regulated gene whose mutation causes disease, *Mmadhc*, methylmalonic aciduria (cobalamin deficiency) cblD type, with homocystinuria [30], was also found in this list.

### Whole genome DNA methylation profiles

**Figure 2:**
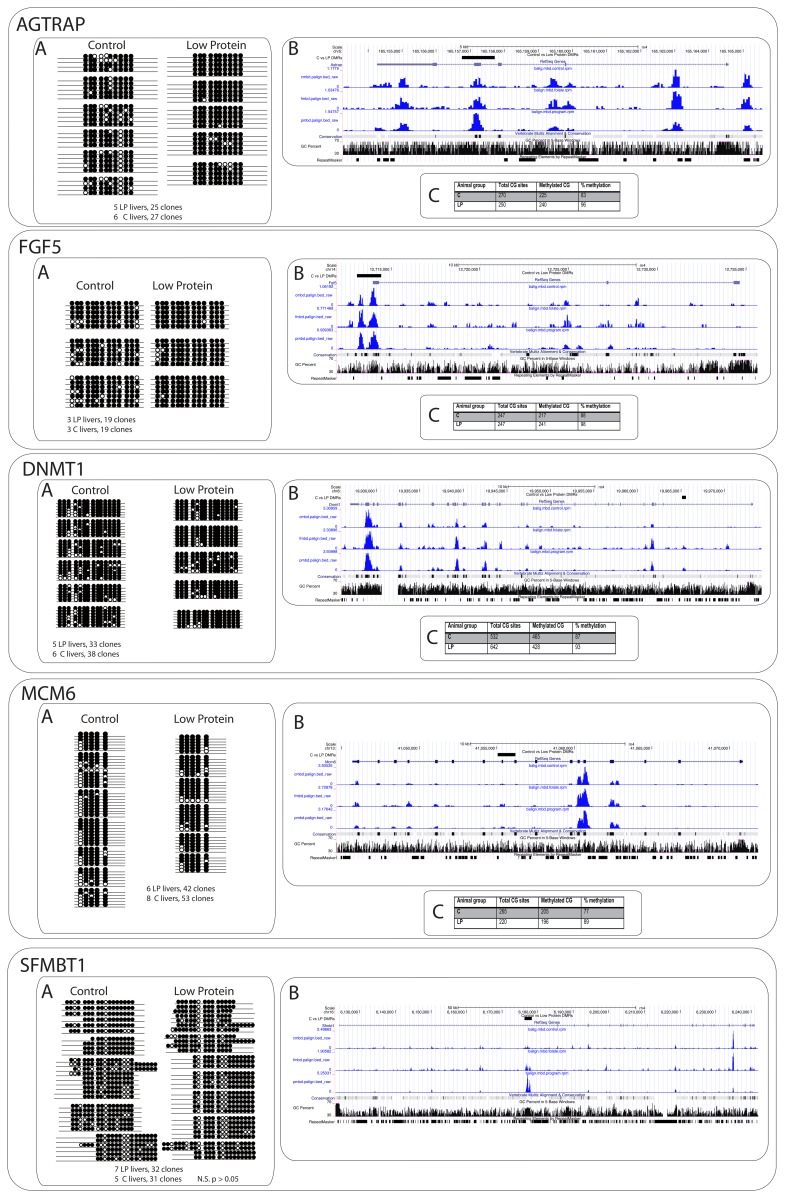
Figure panel representing five genomic loci (*Agtrap, Fgf5, Dnmt1, Mcm6*, S*fmbt*1) containing differentially methylated regions (DMRs) between livers obtained from newborns whose mothers were fed a control diet (18% protein) and livers obtained from newborns whose mothers were fed a low protein (9% protein) diet. For each locus, Panel A shows the validation data obtained from bisulfite conversion followed by cloning and sequencing, which provides methylation status at single CpG site resolution for different livers and different clones (black circles represent methylated CpG, white circles unmethylated CpG). All loci except for *Sfmbt1* validated significant difference in DNA Methylation (*Agtrap* P<0.01, *Fgf5* P<0.01; Dnmt1 P<0.05; *Mcm6* P<0.01, Mann-Whitney U test), in line with that indicated by genome-wide MBD-Seq data. Panel B provides an overview of the genomic locus, the gene structure, the location of the DMR validated, and the level of methylation across the locus for each of the animal groups analyzed (Control = “cmbd”, Low Protein = “pmbd”, and Low Protein with Folic Acid Supplementation = “fmbd”). Panel C summarises the percent methylation in control and low protein for each locus assessed by bisulphite sequencing.

Genomic DNA from the samples was subjected to selection according to the methyl-CpG binding domain-based (MBD) protein protocol (DNA pooled from 8 individual animals per group). The DNA fragments so selected were then subjected to high throughput sequencing using Illumina Genome Analyzer II technology. The resulting DNA sequences were mapped to the rat genome assembly Nov 2004 (Baylor 3.4/rn4) using Bowtie [31], and the resulting alignments were normalized to create methylation tracks (see *Supplementary Data*), which were subsequently visualised in UCSC Genome Browser. We finally modelled the DNA epigenetic marks using MACS [32] (see details in Materials and Methods). Comparing MLP to C, we selected those differentially methylated regions (DMRs) that exhibited calculated FDR = 5%. Therefore, out of a larger (FDR = 10%) collection of differentially methylated regions (see relevant GEO dataset), 1183 genomic loci were retained for downstream analysis in MLP livers in comparison to controls. Hypermethylated regions identified comparing MLP to C livers had an average size of ∼800 bp, a mean fold enrichment of ∼8, and average distance from the Transcription Start Site (TSS)∼80,000 bp. Detailed statistics of these features as well as fold enrichments is provided in *Table S1*. These regions were subsequently annotated to the ‘nearest gene’ using Ensemble API as described in Materials and Methods (DATASET S6). Genomic annotation indicated that these regions were distributed both within genes (40%) and between genes (58%) with 2% overlapping either exons or 5′/3′UTRs (*Table S1*). A large number of DMRs also mapped to repetitive elements within the genome. No single class of repeat significantly outnumbered others, but they included Line 1 repeats (70), dust (42) and trf (29) repeats (*Table S1*). Differentially methylated loci annotated to *Dnmt1*, *Agtrap*, *Fgf5*, and *Mcm6* were validated by conventional bisulphite sequencing, confirming the genome-wide results (one region annotated to Smfbt1 could not be validated, though). The validation data and genomic context of these DMRs is shown in Figure 2. The total number of unique gene symbols that bore differential DNA methylation at FDR 5% was 630.

This gene list was further investigated using GSEA-MSigDB [23], [24]. Interestingly, we found that a large subset of these DMR-neighbouring genes was associated with down-regulation in an experiment with UV-induced mutation of *ERCC3* transcription factor [33], as well as with a distinctive signature for *MLLT3* (*FOXO* transcription factor) in the regulatory regions (within 2 Kbp from the TSS) of more than one hundred genes. The complete analysis can be found in DATASET S7. No enrichment in miRNA signatures was observed, which was subsequently confirmed by GREAT [34]; and no enrichment in canonical pathways or GO terms could be detected. Therefore, we also performed functional enrichment analysis via GREAT [34], using mouse and human orthologous regions for the DMRs that we found in rat (see Materials and Methods). The functional enrichment analysis overall pointed to immune response, especially to the function of the immune component that fights infection and which is also involved in autoimmune diseases.

Using mouse regions and annotating to the ‘nearest gene’ GREAT rule, enrichment in functions related to the ‘*activation of the immune response*’ and ‘*positive regulation of the immune system process*’ as well as ‘*differentiation of retina/eye photoreceptor*’ (corrected p-values ranging between 10^−3^–10^−2^) was found. Enrichment in ‘Mouse phenotype’ terms relevant to ‘*abnormal CD8-positive T cell morphology/number*’, ‘*abnormal single-positive T cell number*’ and ‘*decreased single-positive T cell number*’ (corrected p-values 10^−2^), suggests deregulation of this immune system component. Using human regions, no enrichment in GO terms was found; yet the same enrichment in ‘Mouse phenotype’ terms was reproduced. A few additional enriched terms in this collection pointed to developmental functions (DATASET S7). Performing the same analysis with GREAT extension rules (see Materials and Methods), for both human and mouse orthologs regions lists, we found again that several immune system functions were enriched (corrected p-values∼10^−2^) (DATASET S7). Adopting a wider annotation rule allowed us to identify further immune signatures such as those involving CD4+ T-cells and innate immune system. Overall, regardless of the species and rule used for annotation, immune system enrichment was evident. This seemed to correlate well with the above-mentioned ‘FOXO signature’ discovered with GSEA-MSigDB. FOXO proteins are important for developmental regulation, for example in the differentiation of T-cells to become regulatory T cells [35] (see discussion).

Comparing MLP-F to C no differentially methylated regions were found, possibly indicating a protective action of folate supplementation against the MLP effect on DNA methylation. Once more assuming reversal upon folate supplementation, we compared MLP to MLP-F livers. We obtained 35 hypermethylated regions at FDR 5% subsequently annotated using Ensemble API, which (partially) overlapped the best differentially methylated regions identified in comparison MLP vs. C (see DATASET S8). These DMRs mapped to 17 unique gene symbols amongst which *Sfmbt1* (*polycomb*) and *Acadm*, an enzyme whose deficiency causes serious hepatic dysfunction. Approximately half of these 35 loci bore either no gene symbol or their relevant identifier could not be mapped to ontological collection, and no functional enrichment could be detected. Although 14 DMR loci from both lists perfectly overlapped at FDR 5%, we noticed that many more DMRs could be identified in MLP vs. C as compared to MLP vs. MLP-F, suggesting that the changes induced by the folic acid do not completely rescue the disturbance. This discordance may potentially be related to the ‘digital’ nature of the technology employed for detecting DMRs, and would also require investigation into dosage effects and sample variability.

### GWAS and DNA methylation

We evaluated the overlap between differentially methylated regions in MLP vs. C (rat) and loci found in GWAS studies (human) pertaining to metabolic and cardiovascular disorders (diabetes, high blood pressure, cardiovascular function, and obesity), using their associated gene lists. The overlap with 104 GWAS genes was found to be statistically significant (hypergeometric probability p = 0.0025). This set included genes (see Table 1) such as *FTO*, for which a link between genetic state (SNP allele, within an intron) and DNA methylation has been published previously [36], and *BACH2*, a transcription factor which maintains immune homeostasis [37] and whose polymorphisms have been associated with several inflammation-related autoimmune diseases. The statistical significance of this overlap, linked to the size of gene lists, may vary according to how the GWAS lists were compiled. Nevertheless, in the light of recent discoveries [38], [39], this finding suggests potential, dysfunctional interplay of genetic and epigenetic alterations, implicating DNA methylation disturbance.

**Table 1:** List of loci from selected GWAS studies pertaining to metabolic and cardiovascular phenotypes associated to Low Protein diet that contain differentially methylated regions in their orthologous rat locus.

GWAS Locus	Disease
BACH2	Type 1 Diabetes
CTLA4	Type 1 Diabetes
CTSH	Type 1 Diabetes
PTPN2	Type 1 Diabetes
AGTRAP	Systolic Blood pressure
ARID5B	Diastolic Blood pressure
PRDM8	Diastolic Blood pressure
SETBP1	Ventricular Conduction
VTI1A	Ventricular Conduction
FTO	Obesity and Obese Type 2 Diabetes

The overlap between the total number of GWAS loci investigated and DMR containing loci is significant (p = 0.0025).

### Gene expression and DNA methylation

**Figure 3:**
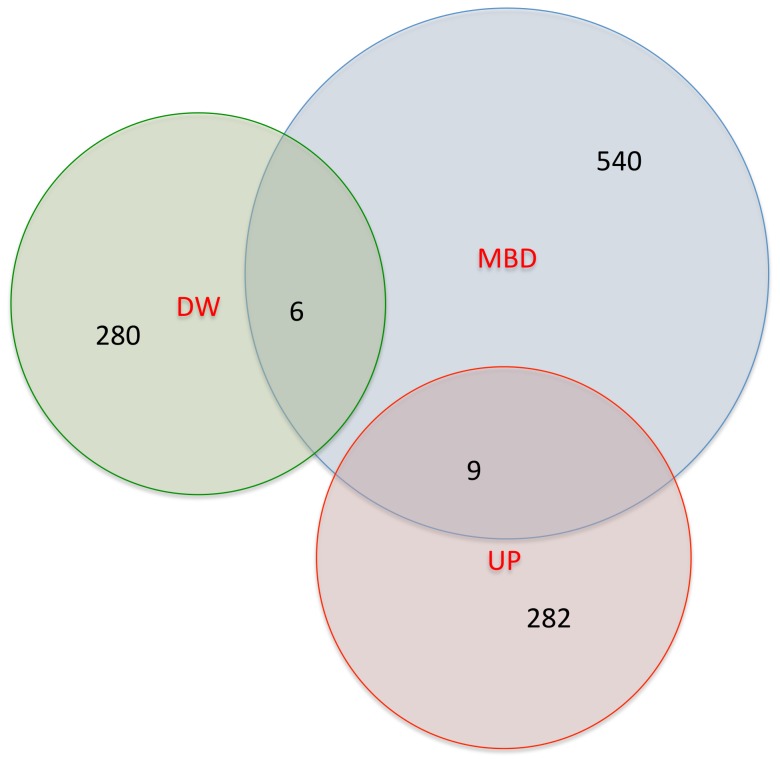
Intersection of differentially expressed (GEX), separated in up-regulated (UP) and down-regulated (DW), and DMRs-neighboring (MBD) genes. At FDR 5% only 15 genes (hypergeometric probability = 0.69) are found in both lists, of which 6 were down-regulated (*Dym*, *Glrx2*, *Mlph*, *Myh10*, *Pftk1*, And *Prep*) and 9 up-regulated (*Abcc8*, *Abhd6*, *Cep350*, *Ctsa*, *Eif2b3*, *Pdk1*, *Serpina11*, *Tpcn2*, And *Tyw3*). The total number of mapped genes is 555 in MBD and 577 in GEX.

There was no significant overlap between genes associated with differentially methylated regions and differentially expressed genes, as shown in Figure 3. Only 9 up-regulated genes and 6 down-regulated genes were found at the intersection with DMR-neighbouring genes (both lists taken at FDR 5%; hyper-geometric probability = 0.69). The DNA methyltransferase-1, *Dnmt1*, which was down-regulated (see Figure 1B) and bore a validated (intronic) DMR at FDR higher than 5%, does not appear in this list (see discussion). The functions of these 15 genes can be found in Figure S1, along with relevant genomic details. Functional enrichment analysis of the 15 DMRs-neighbouring genes did not highlight common pathways, and no correlation of up/down-regulation with the genomic context of DMRs could be determined. Ten of these 15 methylation marks occurred in introns, which traditionally is associated with gene silencing [40], [41]. However, only 4 of these marks were linked to down-regulated genes (*Dym, Glrx2, Myh10, Pftk1*, see Figure S1). Additionally, comparative analysis of DMR-neighboring and differentially expressed gene sets (FDR 5%) with GENEGO showed a striking inverse relationship in 3 categories: ‘*cell cycle and its regulation*’ function (highly enriched in GEX and low in MBD), ‘*apoptosis*’ and ‘*mitogenic signaling*’ (highly enriched in MBD and low in GEX (see Figure S2). This discordance, though not completely unexpected (see discussion), argues for a greater understanding of the complex interplay between DNA methylation and gene expression in fetal programming models, across tissues and development.

## Discussion

The phenomenon of fetal programming by which intrauterine stressor are associated with physiological and metabolic effects in adult offspring is now well established in a variety of species, and good evidence indicates that this contributes to human disease. Such observations highlight the importance of the intrauterine environment for human health with substantial public health implications. The mechanisms that underlie this phenomenon are less clear, but accumulating evidence supports the “DNA methylation hypothesis” [42]. In summary, this hypothesis suggests that since that intrauterine life coincides with a period of major epigenetic modification of the fetal genome, and since many of these DNA methylation changes are highly stable, disturbance of this epigenetic process through adverse environmental exposure has the potential to influence gene expression throughout the life of the offspring. There are numerous examples from animal models of undernutrition in pregnancy which indicate effects of the nutritional environment upon specific gene promoters in specific tissues [6]. Similarly, differential methylation has been observed in the *IGF-2* gene in humans who were exposed to the 1944/5 Dutch famine *in utero* [43]. The primary aim of this study was to establish whether maternal dietary effects upon DNA methylation and gene expression occur across the whole genome. Our findings show an excellent correlation of such genome-wide patterns with relevant phenotypes, although arguing for a greater understanding of the molecular basis of fetal programming.

It is clear that a multitude of anatomical and physiological processes are altered in models of fetal programming and that these stem from underlying alterations in gene expression [44], [45]. An undoubted difficulty with such observations is to distinguish primary alterations in gene expression from secondary alterations occurring consequent to primary alterations. For example the profound hepatic steatosis developed in aged rats exposed to protein restriction *in utero* is associated with hepatic over-expression of *SREBP1c* and the lipogenic pathway [44]. However in younger animals the same pathway is suppressed and there is no evidence of differential expression at birth or during the fetal period [27], [46]. Furthermore, it is clear that increased global DNA methylation is present in some tissues in rodent models of nutritional programming, and differential methylation in a number of specific genes and specific sites within these genes has been described and correlates, in published examples, with the observed gene expression changes [12], [14].

In this study we have used a well-established model of fetal programming in the rat and shown that a maternal low protein diet is associated with a significantly disturbed pattern of altered gene expression in the liver from as early as P1. Others have shown that this, or a similar model is associated with altered hepatic gene expression by 4, 8 or 12 weeks of age, with prolonged effects into ageing, and thus this finding is not unexpected. Indeed we have recently shown that protein restriction and maternal iron deficiency disturb similar gene pathways (cell cycle regulation, mitosis) as early as d13 gestation [45], [47]. The ontogeny of the differentially expressed genes differs in later time point experiments from those observed at P1. This should not be unexpected as obvious physiological and nutritional developments occur between neonatal and more mature animals. These are inevitably accompanied by maturation in gene expression patterns in both the liver and elsewhere. Secondary changes in gene expression will occur at later time points, particularly in response to disturbed physiological and metabolic phenotypes, as we observed in earlier work [27], [29], [48]. Transient disturbance of expression may result from short-term consequences of the adverse maternal environment.

A substantial number of differentially methylated regions were observed in comparing MLP and control animals by whole genome MBD-Seq analysis. These were widely distributed across the genome and did not exhibit strong relationships with gene promoters or other recognised gene regulatory regions, as has been also shown in numerous other genome-wide studies, which have clearly indicated that CpG islands and promoters constitute only a fraction of the genome affected by DNA methylation [49]. There was a high frequency of association with a variety of types of DNA repeat, the significance of which is not clear, although this phenomenon has been observed in other models of differential DNA methylation [36]. We validated DMRs at a greater FDR than the one used for the annotation, so it is possible that further biologically relevant enrichments would be identified if we utilized a wider set of differentially methylated regions. Nevertheless, DNA methylation, although assessed using a single molecular assay, reflects a multitude of functional mechanisms in relation to the location of the signal (gene body, intergenic regions, promoters, etc). In absence of dedicated annotation tools, we have employed two different annotation methodologies in a complementary way. The pathways so identified (immune/developmental signatures) are consistent, yet further validation would be required.

It is notable that a large subset of DMRs-neighbouring genes were found sharing binding sites that match annotation for *FOXO* (or *MLLT7*, the *human homolog of Drosophila trithorax MLL*), a negative regulator of insulin sensitivity in liver, adipocytes and pancreatic cells [50], which may suggest that these genes are somehow functionally related, perhaps through *trithorax*. Significant association with *AR* and *AP2* response elements in up-regulated genes as well as with liver-specific functions was observed. Furthermore, down-regulation at P1 was very significantly associated with *E2F* binding sites in a subset of genes, including strong downregulation of *Ezh2* and *H2afz* (*polycomb*). Notably, conditional deletion of *Polycomb* protein *EZH2* in mouse *beta*-cell determines *beta*-cell regeneration failure leading to diabetes mellitus [51].

Having established the concomitant presence of differential gene expression and differential DNA methylation, it might be assumed in the light of previously published work that the genes affected would be largely the same. As is readily apparent from Figure 3, this was clearly not the case. Only 3 of the genes that are differentially expressed at FDR 1% were also associated with DMRs (and 15 genes at FDR 5%). Such discordance is consistent with other recent studies [52], [53]. To identify more substantial concordance between gene expression and DNA methylation signatures would probably require the use of specific tissues, time points and possibly environmental stimuli that would elicit a gene expression outcome from loci epigenetically programmed during development. The apparent discordance between the lists of DMRs-neighboring and differentially expressed genes may also be explained by differential methylation essentially only impacting upon expression under conditions of stimulus or challenge, e.g. in response to endocrine signals, or to metabolic stressors, that are absent in the P1 liver. One alternative model is that in which an unknown factor results in both altered DNA methylation and gene expression. This alternative factor may be early stable histone modifications such as those resulting from *polycomb/trithorax*-type developmental influences [54]. The finding of a close relationship between *E2F* recognition elements and genes showing altered expression is compatible with this. Notably, *E2f7* and *E2f8* were respectively methylated and differentially expressed (down-regulated) in MLP vs. C.

It was interesting to discover a significant overlap between DMRs and loci involved in GWAS studies for metabolic disorders such as type 1 diabetes, obesity and blood pressure. This was particularly remarkable given that this comparison involves comparing genetic and epigenetic loci as well as comparing human studies to animal model studies. Although the importance of this overlap needs confirmation through further studies, the observation strongly supports the hypothesis that complex traits and diseases are under the control of specific loci both genetically and epigenetically. It would also suggest that fetal programming effects might be linked to modulation of the epigenetic state of loci that are related to disease at a genetic level. Improved predictive ability might therefore develop from consideration of both the genetic and epigenetic state of a locus.

The observation that folic acid supplementation prevented or reversed both the changes in gene expression, (in keeping with observations at later time points of development [55]) and the changes in DNA methylation is of considerable interest. A general disturbance of 1-carbon metabolism associated with maternal protein restriction (suggested by the dysregulation, rescued by folate, of *Gnmt* in MLP), is one candidate mechanism that may explain this. However, whilst there have been reports of elevated maternal plasma homocysteine concentrations in protein restriction [18] there is no convincing metabolic data that suggests the protocol leads to folate insufficiency or gross changes in the methionine-homocysteine, or folic acid cycles [16]. The observation that maternal low protein feeding down-regulates expression of *Dnmt1* in the offspring has been previously reported in older animals, suggesting that the effect lasts until at least weaning [12]. The effect of folate is more challenging to explain, but could be related to flux through the methionine-homocysteine pathway and the inhibitory effect of S-adenosylhomocysteine on *Dnmt1* [56], [57].

## Conclusions

In summary, this study has provided evidence of a substantial effect of diet during pregnancy on both the gene expression and DNA methylation genome-wide profile in newborns. The loci affected in terms of gene expression and DNA methylation were quite different, probably reflecting different roles of these signatures. Whilst we noted some evidence that diet impacted upon expression and epigenetic regulation of *Dnmt*s, inconsistency and subtle variations in the data suggest more intricate mechanisms are involved in the response to early diet. There was a significant overlap between loci affected in terms of DNA methylation in the model, with loci from GWAS studies for metabolic and cardiovascular disorders. Together these findings indicate that improved understanding of complex traits and diseases is likely to require further integration of human and animal studies, with amalgamation of genetic, epigenetic, and gene expression-based approaches.

## Materials and Methods

### Ethics statement

All animal experiments were performed in the BioResources Unit of the University of Nottingham, under license from the United Kingdom Home Office in accordance with the 1986 Animals (Scientific Procedures) Act. The study was approved by the UK Home Office (Project License PPL40/2990) and University of Nottingham Ethics Committee (approval ID SLE/005/07).

### Animals

Virgin female Wistar rats (Harlan, UK) were mated at between 180 and 225 g weight, to a single stud male. Upon confirmation of pregnancy by the presence of a semen plug the female rats were randomly allocated to be fed one of three synthetic diets (18% casein, 1 mg/kg folate Control; 9% casein, 1 mg/kg folate MLP; 9% casein, 5 mg/kg folate MLP-F). See Table S2. The full composition of the diets is published elsewhere (14). Pregnant dams were fed the semi-synthetic diets until they delivered pups at 22 d of gestation. At 1 day after birth, the male pups were culled by cervical dislocation. Liver tissue was collected, snap-frozen in liquid nitrogen and stored at −80°C. One litter from each group was studied, generating up to 16 offspring in each case. DNA was pooled from livers of 8 individual animals per group. Four of these livers also provided samples for RNA preparations.

### Microarray experiment

Total RNA were isolated from three groups of 1 day male rat livers using RNeasy mini kit (QIAGEN), treated with Turbo DNaseI (Ambion) for 30 min at 37°C, then with phenol/chloroform and ethanol precipitated. The quality of RNA was validated by an Agilent 2100 Bioanalyzer (Agilent Technologies). Four RNA samples for each group were hybridized to RatRef-12 Expression BeadChip Microarray (Illumina). All samples exhibited signal-to-noise ratio above 11. Raw data was analyzed using *beadarray* and *limma* R-Bioconductor packages (see links below). Quantile normalization, shrinkage (eBayes) and Benjamini-Hochberg false discovery rate control methods (FDR) were devised. Heatmap and volcano plot for comparison MLP/C are shown in Figure S3 and Figure S4 respectively. GEO accession number of this experiment is GSE50799.

### Validation of gene expression data

1 µg of RNA was reverse transcribed with SuperScriptII (Invitrogen) and used as a template in real-time q-PCR. All PCR amplifications were performed using a Kappa SYBR FAST qPCR master mix kit (Kappa Biosystems). Cycling conditions: denaturation at 95°C for 3 min, then 40 cycles of 95°C for 3 sec, 60°C for 20 sec, followed by dissociation curve step. All real-time PCRs were carried out using the Mx3000 Multiplex Quantitative PCR System (Stratagene). All reactions were performed twice in triplicate. Reaction specificity was verified by melting curve. Primers were designed to amplify 8 genes: *Dnmt1*, *Dnmt3a*, *Dnmt3b*, *AurKB*, *Mcm6*, *Hat1*, *Gnmt* and *Gmnn*. Primer sequences are listed in *DATASET S9*. P-values (Student's t-test) less than 0.05 were deemed significant (see Figure 1B).

### MBD enrichment of methylated DNA

Genomic DNA was isolated using QIAamp DNA mini kit (QIAGEN) and fragmented by sonication to 500 bp using the Bioruptor (Diagenode). His_6_-GST-MBD fusion protein was incubated with 1 µg of fragmented DNA (for 1 reaction) for 2 hours at 4°C, followed by incubation with magnetic beads for another hour at 4°C. Methylated DNA was eluted from beads-MBD-DNA complexes according to the manufacturer's instructions (MethylCap kit, Diagenode). Eluted fractions were purified through the spin columns (QIAGEN, PCR purification kit) and stored frozen. High salt fraction was used for library preparation. MethylCap enrichment efficiency in the same IP sample was calculated by Q-PCR comparison of the specific gene and non-methylated DNA using rat meDNA primer pair (TSH2B) and rat unDNA primer pair (GAPDH) (Diagenode). DNA was pooled from 8 individual animals per group (same litter).

### Library preparation

Approximately 10 ng of captured DNA was used for library preparation (ChIP-seq DNA sample Prep Kit, Illumina). DNA was first end repaired, and then A-overhangs were added to the 3′ ends. After ligation of Illumina adapters 300 bp DNA was isolated by 2% NuSieve 3∶1 agarose (LONZA) gel electrophoresis, gel purified at RT, eluted from the columns (QIAGEN) and enriched by 18 cycles of PCR. The library was evaluated using an Agilent 2100 Bioanalyzer (Agilent Technologies), quantified by q PCR with Illumina primers followed by paired –end 76-bp sequencing using an Illumina Genome Analyzer II according to the manufacturer's instructions in Genome Centre. GEO accession number of this experiment is GSE50935.

### MBD sequencing quality control

Quality control of the sequenced libraries was performed using FastQC software (see *web links* below) as well as in-house scripts in order to provide complementary information whenever necessary. FastQC assessed six features for each (76 bp) read in each library as follows: *per base* sequence quality, *per sequence* quality scores, *per base* sequence content, *per base* GC content, *per sequence* GC content, *per base* N content, sequence length distribution, sequence duplication levels, overrepresented sequences, presence of contaminants (K-mers level). Further quality control was performed after aligning the reads to the genome (see below).

### Pair alignments

Alignments to the reference genome, *Rattus Norvegicus* rn4, were performed using Bowtie [31] and retaining one alignment per read pair. In case of multiple locations, either the alignment with highest score was retained or the read pair was definitely discarded. The alignments were also normalized and transformed in genomic tracks for visualization in UCSC genome browser (see *web links* below) using the R package MEDIPS [58]. The same package was also used to evaluate coverage, saturation and GC content of our MBD-seq data. This step completed our quality control procedure. Only libraries that passed all quality control steps were further processed. Library saturation levels for C, MLP and MLP+F were (as measured by Pearson's coefficient) 0.98, 0.99 and 0.99, respectively. *See also Table S3*.

### Differentially methylated regions

Regions of signal variability were identified using MACS [32], a model-based method developed for analyzing ChIP-seq data, given the many analogies between MBD-seq and ChIP-seq. Differentially methylated regions in comparison MLP vs C and C vs MLP; MLP+F vs C and C vs MLP+F; MLP vs MLP+F and MLP+F vs MLP were analyzed. After a sensitivity study, all adjustable parameters were set to default values whenever possible. Effective genome size was set to 2.00e+09 bp, model fold to 32, p-value cut-off to 10^−5^ and bandwidth to 300 bp. The hypermethylated regions as well as methylation tracks were visualized in UCSC genome browser (see *web links* below). See also Table S1 and Table S3.

### Bisulphite sequencing

1 µg of genomic DNA from each liver sample was bisulphite converted according to manufacture's instruction (EZ DNA methylation Gold kit, ZYMO Research). Converted DNA was eluted from the column with 12 µl of elution buffer. 2 µl of this DNA was used as a template for the 1^st^ round of PCR that were separated by gel electrophoresis, gel purified, eluted in 50 ul of EB, then 4–10 µl of this was used for 2^nd^ round PCR (in total volume 25 µl) with forward nested primer. The amplified products were gel purified and cloned into pCR-4 TOPO vector (TOPO TA Cloning kit, Invitrogen). Primers for PCR were designed to amplify DMR of the rat *Dnmt1*, *Agtrap1*, *Mcm6*, *Fgf5* and *Sfmbt* genes. Primer sequences are listed in *DATASET S10*. Individual clones were analyzed for the presence of cloned DNA fragment by colony PCR. Plasmid DNA was isolated from correct clones (Qiagene). For each animal group up to 6 livers and 53 clones were sequenced using T3 sequencing primer (see detail in DATASET S10). To evaluate the significance of methylation enrichment, U-statistics was applied (DATASET S11).

### Genomic annotation

The differentially methylated regions were annotated by (a) querying Ensembl Rattus Norvegicus repository database for ‘*nearest genes*’ and ‘*nearest repeat*’ up to 10 Kb from annotated TSS via in-house script based upon the Application Programming Interfaces provided by Ensembl API Core (see *web links* below); (b) using GREAT [34], after applying UCSC Utility batch coordinates conversion (see web links below) to convert the rat genomic regions identified via MBD-seq to Mouse and Human orthologs (the two closest species of the three supported at the moment). GREAT employs three annotation rules as follows: ‘nearest genes’ (directly comparable to our in-house annotation above), ‘basal+ extension’ and ‘two nearest genes’.

### Ontological annotation

All gene lists were annotated by employing GSEA-MSignDB [23], [24] and the proprietary software GENEGO from Metacore web portal (see web links below). GENEGO was also utilized for performing the mutual comparison of gene expression and DMR-neighboring gene lists. Additional annotation was performed using GREAT, as specified above. The lists of gene symbols used as input can be found in Datasets D2, D4, D5 and D7. The p-values for overlaps of experimental lists with standard biological collections (BioCarta, Kegg, Reactome and GO) and differentially expressed gene lists from MSignDB collections were generated by hypergeometric tests implemented in GSEA-MSignDB toolkit, as described in [23], [24] and GSEA website. All gene collections can be inspected and are available in interactive manner at GSEA web site (see link below). Hypergeometric probability for overlaps of experimental lists as well as overlaps of experimental lists and GWAS list were computed using an *in-house* script and gene universe size = 15,000; and discussed with reference to p-value cutoff 0.05. GREAT p-values are calculated using binomial test as described in [34] and relevant website (see links below).

### Web links

Beadarray http://www.bioconductor.org/packages/2.12/bioc/html/beadarray.html

Limma http://www.bioconductor.org/packages/2.12/bioc/html/limma.html

FastQC: http://www.bioinformatics.babraham.ac.uk/projects/fastqc/

UCSC genome browser: http://genome.ucsc.edu/

UCSC LiftOver: http:// genome.ucsc.edu/cgi-bin/hgLiftOver

Ensembl API Core: http://www.ensembl.org/info/docs/api/core/index.html#api

GENEGO: https://portal.genego.com

GSEA-MSigDB: http://www.broadinstitute.org/gsea/msigdb/index.jsp

GREAT: http://bejerano.stanford.edu/great/public/html/

## Supporting Information

Dataset S1 **PvC GEX output (FDR 5%).** Differentially expressed gene probe set in maternal low protein compared to control.
(TXT)

Dataset S2 **GEX PvC, Ontology.** Ontology of the mapped differentially expressed gene set (maternal low protein compared to control). File C2CGP: Overlap matrix by differentially expressed gene list (vertical) and gene sets (horizontal) that represent expression signatures of chemical and genetics perturbation (CGP) in MSigBD collection C2. File C2DWreg: Overlap matrix by down-regulated gene list (vertical) and gene sets (horizontal) collected from various sources in MSigBD collection C2. File C2UPreg: Overlap matrix by up-regulated gene list (vertical) and gene sets (horizontal) collected from various sources in MSigBD collection C2. File C3DWreg: Overlap matrix by down-regulated gene list (vertical) and gene sets (horizontal) that contain genes that share a conserved cis-regulatory motif in promoters and 3-UTRs in MSigBD collection C3. File C3UPreg: Overlap matrix by up-regulated gene list (vertical) and gene sets (horizontal) that contain genes that share a conserved cis-regulatory motif in promoters and 3-UTRs in MSigBD collection C3. File C5DWreg: Overlap matrix by down-regulated gene list (vertical) and gene sets (horizontal) that are annotated to GO terms in MSigBD collection C5. File C5UPreg: Overlap matrix by down-regulated gene list (vertical) and gene sets (horizontal) that are annotated to GO terms in MSigBD collection C5. File Summary and Description: Extensive description of the gene sets shown in Dataset S2. File ReadMeFile: Instruction on how to reconstitute the ontology workbook from separated text files.
(TXT)

Dataset S3 **PvF GEX output (FDR 5%).** Differentially expressed gene probe sets in maternal low protein compared to maternal low protein supplemented with folic acid.
(TXT)

Dataset S4 **GEX PvF, Ontology.** Ontology of the mapped differentially expressed gene set (maternal low protein compared to maternal low protein supplemented with folic acid). File CP: Results from overlapping differentially expressed gene list and gene sets from KEGG pathway database in MSigBD collection C2. File TFBS: Results from overlapping differentially expressed gene list and gene sets that share a conserved cis-regulatory motif in promoters and 3-UTRs in MSigBD collection C2. File GO-processes: Results from overlapping differentially expressed gene list and gene sets from Biological processes Ontology in MSigBD collection C2.
(TXT)

Dataset S5 **GEX Intersection ontology.** Ontology of the mapped differentially expressed gene set, which are found in both maternal low protein compared to control and in maternal low protein compared maternal low protein supplemented with folic acid. File GeneGo: Results from overlapping differentially expressed gene list and gene sets from Biological processes Ontology in MSigBD collection C5. File GeneList: Symbols and descriptions for the differentially expressed genes in both group comparisons. File Pathways: Results from overlapping differentially expressed gene list and gene sets from Canonical Pathway in MSigBD collection C5.
(TXT)

Dataset S6 **PvC MBD differential methylation-** ***annotated*** **, FDR 5%.** Differentially methylated regions-associated gene set in maternal low protein compared to control.
(TXT)

Dataset S7 **DMR-neighbouring genes (MBD-Seq), Ontology, PvC.** Ontology of the mapped differentially methylated regions-associated gene set (maternal low protein compared to control). File C2CGP: Overlap matrix by differentially expressed gene list (vertical) and gene sets (horizontal) that represent expression signatures of chemical and genetics perturbation (CGP) in MSigBD collection C2. File C3: Assuming an hypothetical differential expression for the DMR-neighbouring genes, overlap matrix by this gene list (vertical) and gene sets (horizontal) that contain genes that share a conserved cis-regulatory motif in promoters and 3-UTRs in MSigBD collection C3. File Summary and Description: Extensive description of the gene sets shown in Dataset S7. File outputgreat H19 basal+extension: Results from annotating human orthologs regions with GREAT rule basal+extension. File outputgreat H19 nearest: Results from annotating human orthologs regions with GREAT rule nearest gene. File outputgreat H19 two nearest: Results from annotating human orthologs regions with GREAT rule nearest two genes. File outputgreat MM9 basal+extension: Results from annotating mouse orthologs regions with GREAT rule basal+extension. File outputgreat MM9 nearest: Results from annotating mouse orthologs regions with GREAT rule nearest gene. File outputgreat MM9 two nearest: Results from annotating mouse orthologs regions with GREAT rule nearest two genes.
(DOCX)

Dataset S8 **PvF MBD differential methylation-** ***annotated*** **, FDR 5%.** Ontology of the mapped differentially methylated regions-associated gene set (maternal low protein compared to maternal low protein supplemented with folic acid).
(TXT)

Dataset S9 **Primers for GEX validations.** The primers used for validation of differentially expressed gene probes in both maternal low protein compared to control and in maternal low protein compared maternal low protein supplemented with folic acid.
(DOCX)

Dataset S10 **Primers for MBD validations.** The primers used for validation of differentially methylated regions in maternal low protein compared to control, as illustrated in panel A of *Figure 2* *.*
(DOCX)

Dataset S11 **U-statistics for BS-Seq validations.** Statistical evaluation of the differential CGs methylation depicted in panel A of Figure 2. File Agtrap1: calculations for region annotated to Agtrap1. File Dnmt1: calculations for region annotated to Dnmt1. File Fgf5: calculations for region annotated to Fgf5. File Mcm6: Calculations for region annotated to Mcm6. File Sfmbt1: calculations for region annotated to Sfmbt1.
(TXT)

Table S1 **Stats of MBD regions, PvC.** Descriptive statistics of hypermethylated regions, which are found in maternal low protein compared to control and the relevant numbers of Human and Mouse orthologous regions employed in ontological analysis.
(DOCX)

Table S2 **Diet-phenotype association.** Description of the dam diets and of the pup phenotypes associated with dam diets. Folic acid supplementation (extra folate) = 4 mg/kg. Diets are matched for energy. No evidence of fetal programming due to high COH.
(DOCX)

Table S3 **Number of total and unique reads per library.** Number of total and unique reads per library.
(DOCX)

Figure S1 **15 DEX genes that bear DMRs.** Genomic details and functional information for 15 differentially expressed genes bearing methylation marks (DMRs) in Maternal Low Protein compared to Control at P1 (FDR 5%). Up/Down-regulation is indicated by red and green highlights respectively. The overlap between the total number of mapped DMRs-neighboring genes and the total number of differentially expressed genes was not statistically significant (p = 0.69).
(TIFF)

Figure S2 **GENEGO picture, Word file with color keys.** Comparative ontological analysis of DMR-neighboring and differentially expressed gene sets, which are respectively represented in blue and orange in the bar chart.
(TIFF)

Figure S3 **GEX quality control: heatmap, PvC.** Heatmap of the top highly expressed gene probes, average signal after filtering low quality probes. Diet of control C represented by green bars, MLP+F group by blue bars, and MLP diet group by red bars.
(TIFF)

Figure S4 **GEX quality control: volcano plot, PvC.** Volcano plot of the differentially expressed gene probes in comparison to C of programmed group P = MLP. The top 200 highly significant gene probes are depicted with red dots. Vertical red lines correspond to fold changes equal to +1 and −1 (Log FC = +−0.01) and horizontal green lines corresponds to probability 0.75 (odds = 3, log-odds = 1.098612).
(TIFF)
