# Supplementary material for: Genome-Wide Methylation and Gene Expression Changes in Newborn Rats following Maternal Protein Restriction and Reversal by Folic Acid
Source: PLoS One. 2013 Dec 31;8(12):e82989. doi: 10.1371/journal.pone.0082989 (PMC3877003; doi:10.1371/journal.pone.0082989)

C2-CGP

overlap matrix by gene and geneset DACOSTA_UV_RESPONSE_VIA_ERCC3_DN [855] DACOSTA_UV_RESPONSE_VIA_ERCC3_COMMON_DN_DN [483] ONDER_CDH1_TARGETS_2_UP [256] CUI_TCF21_TARGETS_2_DN [830] IKEDA_MIR30_TARGETS_UP [116] ZHENG_BOUND_BY_FOXP3 [491] GOBERT_OLIGODENDROCYTE_DIFFERENTIATIONION_DN [1080] MEISSNER_BRAIN_HCP_WITH_H3K4ME3_AND_H3_H3K27ME3 [1069] SCHLOSSER_SERUM_RESPONSE_DN [712] BHAT_ESR1_TARGETS_NOT_VIA_AKT1_DN [88] description

ARID5B X X X X X X AT rich interactive domain 5B (MRF1-like)

DLC1 X X X X X deleted in liver cancer 1

STARD13 X X X START domain containing 13

ITSN1 X X X X intersectin 1 (SH3 domain protein)

FYN X X X X X "FYN oncogene related to SRC, FGR, YES"

DST X X X X dystonin

VPS13A X X X vacuolar protein sorting 13 homolog A (S. cerevisiae)

USP9X X X X "ubiquitin specific peptidase 9, X-linked"

TSPAN5 X X X tetraspanin 5

CHD9 X X X chromodomain helicase DNA binding protein 9

PTPN2 X X X "protein tyrosine phosphatase, non-receptor type 2"

NEK7 X X X X NIMA (never in mitosis gene a)-related kinase 7

PPP2R5C X X X "protein phosphatase 2, regulatory subunit B (B56), gamma isoform"

LHFPL2 X X X X lipoma HMGIC fusion partner-like 2

OGT X X X X O-linked N-acetylglucosamine (GlcNAc) transferase (UDP-N-acetylglucosamine:polypeptide-N-acetylglucosaminyl transferase)

ABI1 X X X X abl-interactor 1

EHBP1 X X X EH domain binding protein 1

TRIM2 X X X tripartite motif-containing 2

SMARCA2 X X X "SWI/SNF related, matrix associated, actin dependent regulator of chromatin, subfamily a, member 2"

RUNX1 X X X runt-related transcription factor 1 (acute myeloid leukemia 1; aml1 oncogene)

PAWR X X X "PRKC, apoptosis, WT1, regulator"

FTO X X X -

PJA2 X X X "praja 2, RING-H2 motif containing"

NRG1 X X neuregulin 1

TIAM1 X X T-cell lymphoma invasion and metastasis 1

SRPK2 X X SFRS protein kinase 2

STK39 X X "serine threonine kinase 39 (STE20/SPS1 homolog, yeast)"

CEP350 X X centrosomal protein 350kDa

ATP13A3 X X ATPase type 13A3

AKAP9 X X A kinase (PRKA) anchor protein (yotiao) 9

PTK2 X X PTK2 protein tyrosine kinase 2

TRIM44 X X tripartite motif-containing 44

DCUN1D4 X X "DCN1, defective in cullin neddylation 1, domain containing 4 (S. cerevisiae)"

MKLN1 X X "muskelin 1, intracellular mediator containing kelch motifs"

CDK8 X X cyclin-dependent kinase 8

CEP135 X X centrosomal protein 135kDa

SETD2 X X SET domain containing 2

RGL1 X X X ral guanine nucleotide dissociation stimulator-like 1

TFPI X X tissue factor pathway inhibitor (lipoprotein-associated coagulation inhibitor)

PSD3 X X X X pleckstrin and Sec7 domain containing 3

LRRFIP1 X X leucine rich repeat (in FLII) interacting protein 1

SFMBT1 X X Scm-like with four mbt domains 1

ZBTB20 X X X zinc finger and BTB domain containing 20

SYNE2 X X "spectrin repeat containing, nuclear envelope 2"

NAV3 X X neuron navigator 3

DOCK1 X X dedicator of cytokinesis 1

SKAP2 X X src kinase associated phosphoprotein 2

CCND1 X X cyclin D1

INHBB X X "inhibin, beta B (activin AB beta polypeptide)"

EFNA5 X X ephrin-A5

VLDLR X X very low density lipoprotein receptor

GNE X X glucosamine (UDP-N-acetyl)-2-epimerase/N-acetylmannosamine kinase

SNX2 X X sorting nexin 2

TAOK3 X X TAO kinase 3

ATXN1 X X ataxin 1

AP3B1 X "adaptor-related protein complex 3, beta 1 subunit"

ERC1 X ELKS/RAB6-interacting/CAST family member 1

TPST1 X tyrosylprotein sulfotransferase 1

WDFY3 X WD repeat and FYVE domain containing 3

WDHD1 X WD repeat and HMG-box DNA binding protein 1

AHDC1 X "AT hook, DNA binding motif, containing 1"

MN1 X meningioma (disrupted in balanced translocation) 1

ATP2B4 X "ATPase, Ca++ transporting, plasma membrane 4"

SMAD4 X "SMAD, mothers against DPP homolog 4 (Drosophila)"

TEAD1 X TEA domain family member 1 (SV40 transcriptional enhancer factor)

MTR X 5-methyltetrahydrofolate-homocysteine methyltransferase

EML1 X X echinoderm microtubule associated protein like 1

MLLT3 X X X "myeloid/lymphoid or mixed-lineage leukemia (trithorax homolog, Drosophila); translocated to, 3"

ENPP1 X X X X ectonucleotide pyrophosphatase/phosphodiesterase 1

PTGER2 X X X "prostaglandin E receptor 2 (subtype EP2), 53kDa"

SYT11 X X synaptotagmin XI

LTBP1 X X latent transforming growth factor beta binding protein 1

HAS2 X X hyaluronan synthase 2

COL5A1 X X "collagen, type V, alpha 1"

FBLN1 X X fibulin 1

STC2 X stanniocalcin 2

LTBP2 X latent transforming growth factor beta binding protein 2

FBN1 X fibrillin 1

DGKI X "diacylglycerol kinase, iota"

MLPH X melanophilin

PRMT2 X protein arginine methyltransferase 2

PRRX1 X paired related homeobox 1

ZFHX4 X zinc finger homeodomain 4

ARHGAP22 X Rho GTPase activating protein 22

SPOCK1 X "sparc/osteonectin, cwcv and kazal-like domains proteoglycan (testican) 1"

PRR16 X proline rich 16

UBE2V2 X X ubiquitin-conjugating enzyme E2 variant 2

PRKCH X X X "protein kinase C, eta"

PTPRC X X X "protein tyrosine phosphatase, receptor type, C"

GATA3 X X X GATA binding protein 3

DUSP6 X X dual specificity phosphatase 6

PCDH17 X X X protocadherin 17

PDE4D X X "phosphodiesterase 4D, cAMP-specific (phosphodiesterase E3 dunce homolog, Drosophila)"

NCOR1 X X nuclear receptor co-repressor 1

MBNL2 X X muscleblind-like 2 (Drosophila)

SHROOM3 X X X shroom family member 3

CDC14A X X CDC14 cell division cycle 14 homolog A (S. cerevisiae)

ATP10A X X "ATPase, Class V, type 10A"

SHE X X Src homology 2 domain containing E

SH3GLB1 X X SH3-domain GRB2-like endophilin B1

SIRPA X X signal-regulatory protein alpha

LRBA X "LPS-responsive vesicle trafficking, beach and anchor containing"

EPHB1 X EPH receptor B1

TTLL7 X "tubulin tyrosine ligase-like family, member 7"

TANC1 X "tetratricopeptide repeat, ankyrin repeat and coiled-coil containing 1"

SETD7 X SET domain containing (lysine methyltransferase) 7

ERG X v-ets erythroblastosis virus E26 oncogene homolog (avian)

COL4A4 X "collagen, type IV, alpha 4"

DPP4 X "dipeptidyl-peptidase 4 (CD26, adenosine deaminase complexing protein 2)"

CCRN4L X CCR4 carbon catabolite repression 4-like (S. cerevisiae)

C1QTNF7 X C1q and tumor necrosis factor related protein 7

ELTD1 X "EGF, latrophilin and seven transmembrane domain containing 1"

RASA2 X RAS p21 protein activator 2

ODZ2 X "odz, odd Oz/ten-m homolog 2 (Drosophila)"

ARHGAP26 X Rho GTPase activating protein 26

FGD5 X "FYVE, RhoGEF and PH domain containing 5"

CYYR1 X cysteine/tyrosine-rich 1

CTDSPL X "CTD (carboxy-terminal domain, RNA polymerase II, polypeptide A) small phosphatase-like"

GLRX2 X glutaredoxin 2

TTC14 X tetratricopeptide repeat domain 14

ATG10 X ATG10 autophagy related 10 homolog (S. cerevisiae)

SLC38A4 X "solute carrier family 38, member 4"

LRRC28 X leucine rich repeat containing 28

SATB1 X X X special AT-rich sequence binding protein 1 (binds to nuclear matrix/scaffold-associating DNA's)

TBC1D15 X X "TBC1 domain family, member 15"

SLC6A6 X X "solute carrier family 6 (neurotransmitter transporter, taurine), member 6"

B4GALT5 X X "UDP-Gal:betaGlcNAc beta 1,4- galactosyltransferase, polypeptide 5"

SEC23A X X Sec23 homolog A (S. cerevisiae)

LARGE X like-glycosyltransferase

MYH10 X "myosin, heavy chain 10, non-muscle"

CCND2 X cyclin D2

ARID4B X AT rich interactive domain 4B (RBP1- like)

USP47 X ubiquitin specific peptidase 47

PI4K2B X phosphatidylinositol 4-kinase type 2 beta

CDH10 X X "cadherin 10, type 2 (T2-cadherin)"

ABLIM1 X X actin binding LIM protein 1

FOXP1 X X forkhead box P1

UTRN X X utrophin (homologous to dystrophin)

OLIG3 X oligodendrocyte transcription factor 3

MAP3K8 X mitogen-activated protein kinase kinase kinase 8

CTLA4 X cytotoxic T-lymphocyte-associated protein 4

BCL11B X B-cell CLL/lymphoma 11B (zinc finger protein)

TBC1D5 X "TBC1 domain family, member 5"

FAM105A X "family with sequence similarity 105, member A"

ANKH X "ankylosis, progressive homolog (mouse)"

ETNK1 X ethanolamine kinase 1

ST6GAL1 X "ST6 beta-galactosamide alpha-2,6-sialyltranferase 1"

PIAS2 X "protein inhibitor of activated STAT, 2"

NSMCE1 X non-SMC element 1 homolog (S. cerevisiae)

NSMCE2 X "non-SMC element 2 homolog (MMS21, S. cerevisiae)"

RFX3 X "regulatory factor X, 3 (influences HLA class II expression)"

ANKRD55 X ankyrin repeat domain 55

TMEM67 X transmembrane protein 67

PPM1L X X protein phosphatase 1 (formerly 2C)-like

SEMA3F X X "sema domain, immunoglobulin domain (Ig), short basic domain, secreted, (semaphorin) 3F"

H2AFV X X "H2A histone family, member V"

ETS2 X X v-ets erythroblastosis virus E26 oncogene homolog 2 (avian)

HSPA4 X X heat shock 70kDa protein 4

ANK3 X X "ankyrin 3, node of Ranvier (ankyrin G)"

DCC X deleted in colorectal carcinoma

CACNB3 X "calcium channel, voltage-dependent, beta 3 subunit"

PRKAR1B X "protein kinase, cAMP-dependent, regulatory, type I, beta"

PDK1 X "pyruvate dehydrogenase kinase, isozyme 1"

ACSBG1 X acyl-CoA synthetase bubblegum family member 1

SYT1 X synaptotagmin I

PDCD4 X programmed cell death 4 (neoplastic transformation inhibitor)

LDLRAD3 X low density lipoprotein receptor class A domain containing 3

UGT8 X UDP glycosyltransferase 8 (UDP-galactose ceramide galactosyltransferase)

PCDH7 X BH-protocadherin (brain-heart)

LRRN3 X leucine rich repeat neuronal 3

PIGT X "phosphatidylinositol glycan anchor biosynthesis, class T"

MPZL1 X myelin protein zero-like 1

TBC1D22A X "TBC1 domain family, member 22A"

CABIN1 X -

CCNF X cyclin F

SPSB1 X splA/ryanodine receptor domain and SOCS box containing 1

FMN2 X formin 2

BTG1 X "B-cell translocation gene 1, anti-proliferative"

RGS3 X regulator of G-protein signalling 3

FGFRL1 X fibroblast growth factor receptor-like 1

KCNA6 X "potassium voltage-gated channel, shaker-related subfamily, member 6"

TTLL5 X "tubulin tyrosine ligase-like family, member 5"

IRGM X "immunity-related GTPase family, M"

IGSF11 X "immunoglobulin superfamily, member 11"

TBC1D23 X "TBC1 domain family, member 23"

DDHD1 X DDHD domain containing 1

GRID2 X "glutamate receptor, ionotropic, delta 2"

SLC6A1 X "solute carrier family 6 (neurotransmitter transporter, GABA), member 1"

IRX5 X iroquois homeobox protein 5

GRIK3 X "glutamate receptor, ionotropic, kainate 3"

MKX X mohawk homeobox

OPRD1 X "opioid receptor, delta 1"

SLCO5A1 X "solute carrier organic anion transporter family, member 5A1"

NTN1 X netrin 1

SCNN1G X "sodium channel, nonvoltage-gated 1, gamma"

SYT6 X synaptotagmin VI

SLC30A10 X "solute carrier family 30, member 10"

EYA4 X eyes absent homolog 4 (Drosophila)

GPR83 X G protein-coupled receptor 83

FREM2 X FRAS1 related extracellular matrix protein 2

EDN3 X endothelin 3

RIN3 X Ras and Rab interactor 3

MGAT5B X "mannosyl (alpha-1,6-)-glycoprotein beta-1,6-N-acetyl-glucosaminyltransferase, isozyme B"

SLC4A4 X "solute carrier family 4, sodium bicarbonate cotransporter, member 4"

PDZRN3 X PDZ domain containing RING finger 3

CD24 X CD24 molecule

DDIT4L X DNA-damage-inducible transcript 4-like

ZNF536 X zinc finger protein 536

E2F7 X E2F transcription factor 7

S100A11 X S100 calcium binding protein A11

SORCS2 X sortilin-related VPS10 domain containing receptor 2

CD302 X CD302 molecule

PTPN14 X "protein tyrosine phosphatase, non-receptor type 14"

NFE2L3 X nuclear factor (erythroid-derived 2)-like 3

ATP7B X "ATPase, Cu++ transporting, beta polypeptide"

PRDM8 X PR domain containing 8

NFATC2 X "nuclear factor of activated T-cells, cytoplasmic, calcineurin-dependent 2"

HTR1B X 5-hydroxytryptamine (serotonin) receptor 1B

ARHGEF17 X Rho guanine nucleotide exchange factor (GEF) 17

EDARADD X EDAR-associated death domain

HCN1 X hyperpolarization activated cyclic nucleotide-gated potassium channel 1

AIM1 X absent in melanoma 1

CACNA1E X "calcium channel, voltage-dependent, alpha 1E subunit"

ADK X adenosine kinase

RSU1 X Ras suppressor protein 1

ATRNL1 X attractin-like 1

SNRPE X small nuclear ribonucleoprotein polypeptide E

SERTAD2 X SERTA domain containing 2

XPA X "xeroderma pigmentosum, complementation group A"

SCP2 X sterol carrier protein 2

ADH5 X "alcohol dehydrogenase 5 (class III), chi polypeptide"

ACTR2 X ARP2 actin-related protein 2 homolog (yeast)

HIPK1 X homeodomain interacting protein kinase 1

ELOVL5 X "ELOVL family member 5, elongation of long chain fatty acids (FEN1/Elo2, SUR4/Elo3-like, yeast)"

SUMO1 X SMT3 suppressor of mif two 3 homolog 1 (S. cerevisiae)

COPS8 X COP9 constitutive photomorphogenic homolog subunit 8 (Arabidopsis)

BET1 X BET1 homolog (S. cerevisiae)

VDAC3 X voltage-dependent anion channel 3

PSMD1 X "proteasome (prosome, macropain) 26S subunit, non-ATPase, 1"

SMAD1 X "SMAD, mothers against DPP homolog 1 (Drosophila)"

ACADM X "acyl-Coenzyme A dehydrogenase, C-4 to C-12 straight chain"

STRAP X serine/threonine kinase receptor associated protein

LSM6 X "LSM6 homolog, U6 small nuclear RNA associated (S. cerevisiae)"

BTG3 X "BTG family, member 3"

BCAM X basal cell adhesion molecule (Lutheran blood group)

SYTL2 X synaptotagmin-like 2

RERE X arginine-glutamic acid dipeptide (RE) repeats

SNTB2 X "syntrophin, beta 2 (dystrophin-associated protein A1, 59kDa, basic component 2)"

CHRM3 X "cholinergic receptor, muscarinic 3"

MEIS1 "Meis1, myeloid ecotropic viral integration site 1 homolog (mouse)"

GABBR2 "gamma-aminobutyric acid (GABA) B receptor, 2"

SIM2 single-minded homolog 2 (Drosophila)

NTRK2 "neurotrophic tyrosine kinase, receptor, type 2"

PRKCE "protein kinase C, epsilon"

ABCC8 "ATP-binding cassette, sub-family C (CFTR/MRP), member 8"

FAM5C "family with sequence similarity 5, member C"

TBX21 T-box 21

CACNA1B "calcium channel, voltage-dependent, L type, alpha 1B subunit"

ECEL1 endothelin converting enzyme-like 1

PTF1A "pancreas specific transcription factor, 1a"

SORCS1 sortilin-related VPS10 domain containing receptor 1

UCP1 "uncoupling protein 1 (mitochondrial, proton carrier)"

DOK6 docking protein 6

PRDM12 PR domain containing 12

FRMPD1 FERM and PDZ domain containing 1

FBXL14 F-box and leucine-rich repeat protein 14

PRKD1 protein kinase D1

VAV3 vav 3 oncogene

OSBP2 oxysterol binding protein 2

SKAP1 src kinase associated phosphoprotein 1

RFX4 "regulatory factor X, 4 (influences HLA class II expression)"

KLHL13 kelch-like 13 (Drosophila)

CSMD2 CUB and Sushi multiple domains 2

PHKB "phosphorylase kinase, beta"

SEMA4A "sema domain, immunoglobulin domain (Ig), transmembrane domain (TM) and short cytoplasmic domain, (semaphorin) 4A"

BACH2 "BTB and CNC homology 1, basic leucine zipper transcription factor 2"

RRS1 RRS1 ribosome biogenesis regulator homolog (S. cerevisiae)

METTL7A methyltransferase like 7A

SMYD3 SET and MYND domain containing 3

ARID2 "AT rich interactive domain 2 (ARID, RFX-like)"

CLK2 CDC-like kinase 2

PPP1R1C "protein phosphatase 1, regulatory (inhibitor) subunit 1C"

CAMTA1 calmodulin binding transcription activator 1

NR3C2 "nuclear receptor subfamily 3, group C, member 2"

ARHGAP18 Rho GTPase activating protein 18

SOCS1 suppressor of cytokine signaling 1

TMED10 transmembrane emp24-like trafficking protein 10 (yeast)

QDPR quinoid dihydropteridine reductase

KCNJ15 "potassium inwardly-rectifying channel, subfamily J, member 15"

TNFSF10 "tumor necrosis factor (ligand) superfamily, member 10"

TOPORS "topoisomerase I binding, arginine/serine-rich"

ABHD6 abhydrolase domain containing 6

SLCO4A1 "solute carrier organic anion transporter family, member 4A1"

SYT10 synaptotagmin X

LRRC3B leucine rich repeat containing 3B

GRIN2B "glutamate receptor, ionotropic, N-methyl D-aspartate 2B"

VDAC1 voltage-dependent anion channel 1

COX6A1 cytochrome c oxidase subunit VIa polypeptide 1

KCNAB2 "potassium voltage-gated channel, shaker-related subfamily, beta member 2"

PREP prolyl endopeptidase

GABBR1 "gamma-aminobutyric acid (GABA) B receptor, 1"

ACTR10 actin-related protein 10 homolog (S. cerevisiae)

ASPH aspartate beta-hydroxylase

FBXL2 F-box and leucine-rich repeat protein 2

SYN2 synapsin II

FAIM2 Fas apoptotic inhibitory molecule 2

CNTNAP2 contactin associated protein-like 2

KCNIP1 Kv channel interacting protein 1

MAPK10 mitogen-activated protein kinase 10

GPR22 G protein-coupled receptor 22

SEC23B Sec23 homolog B (S. cerevisiae)

NDUFB3 "NADH dehydrogenase (ubiquinone) 1 beta subcomplex, 3, 12kDa"

EIF2B3 "eukaryotic translation initiation factor 2B, subunit 3 gamma, 58kDa"

NMNAT2 nicotinamide nucleotide adenylyltransferase 2

CASP8AP2 CASP8 associated protein 2

DLGAP1 "discs, large (Drosophila) homolog-associated protein 1"

NDUFA8 "NADH dehydrogenase (ubiquinone) 1 alpha subcomplex, 8, 19kDa"

LAMA4 "laminin, alpha 4"

DHX40 DEAH (Asp-Glu-Ala-His) box polypeptide 40

FGF21 fibroblast growth factor 21

PEPD peptidase D

MUM1L1 melanoma associated antigen (mutated) 1-like 1

CALN1 calneuron 1

PHACTR1 phosphatase and actin regulator 1

ROBO2 "roundabout, axon guidance receptor, homolog 2 (Drosophila)"

ZNF704 zinc finger protein 704

TTYH3 tweety homolog 3 (Drosophila)

CROCC "ciliary rootlet coiled-coil, rootletin"

RBM10 RNA binding motif protein 10

SLC25A28 "solute carrier family 25, member 28"

DNAJC3 "DnaJ (Hsp40) homolog, subfamily C, member 3"

CEACAM1 carcinoembryonic antigen-related cell adhesion molecule 1 (biliary glycoprotein)

IDE insulin-degrading enzyme

SYK spleen tyrosine kinase

TRAF3 TNF receptor-associated factor 3

ERGIC1 endoplasmic reticulum-golgi intermediate compartment (ERGIC) 1

ATP11A "ATPase, Class VI, type 11A"

POLR1B "polymerase (RNA) I polypeptide B, 128kDa"

GSR glutathione reductase

ACLY ATP citrate lyase

CAMP cathelicidin antimicrobial peptide

CSF3R colony stimulating factor 3 receptor (granulocyte)

FRMD4A FERM domain containing 4A

COTL1 coactosin-like 1 (Dictyostelium)

GNG10 "guanine nucleotide binding protein (G protein), gamma 10"

ZCCHC9 "zinc finger, CCHC domain containing 9"

MAP3K7 mitogen-activated protein kinase kinase kinase 7

USP48 ubiquitin specific peptidase 48

DCAKD dephospho-CoA kinase domain containing

CD180 CD180 molecule

SNRPB small nuclear ribonucleoprotein polypeptides B and B1

METTL8 methyltransferase like 8

XPOT "exportin, tRNA (nuclear export receptor for tRNAs)"

PPHLN1 periphilin 1

STX16 syntaxin 16

PIGF "phosphatidylinositol glycan anchor biosynthesis, class F"

MYO7A myosin VIIA

CYBASC3 "cytochrome b, ascorbate dependent 3"

PLEKHA5 "pleckstrin homology domain containing, family A member 5"

ODZ3 "odz, odd Oz/ten-m homolog 3 (Drosophila)"

DYNLL2 "dynein, light chain, LC8-type 2"

DIP2A DIP2 disco-interacting protein 2 homolog A (Drosophila)

CYP19A1 "cytochrome P450, family 19, subfamily A, polypeptide 1"

SREBF2 sterol regulatory element binding transcription factor 2

MAP2K6 mitogen-activated protein kinase kinase 6

PPARD "peroxisome proliferative activated receptor, delta"

RIC8B resistance to inhibitors of cholinesterase 8 homolog B (C. elegans)

CTDP1 "CTD (carboxy-terminal domain, RNA polymerase II, polypeptide A) phosphatase, subunit 1"

CD109 CD109 molecule

SDK2 sidekick homolog 2 (chicken)

TPK1 thiamin pyrophosphokinase 1

MATN3 matrilin 3

MID2 midline 2

SLC37A1 "solute carrier family 37 (glycerol-3-phosphate transporter), member 1"

NHLRC2 NHL repeat containing 2

LAPTM4A lysosomal-associated protein transmembrane 4 alpha

DPY19L3 dpy-19-like 3 (C. elegans)

DPYSL4 dihydropyrimidinase-like 4

INCENP inner centromere protein antigens 135/155kDa

PDIA5 "protein disulfide isomerase family A, member 5"

EGLN1 egl nine homolog 1 (C. elegans)

TMEM117 transmembrane protein 117

SETBP1 SET binding protein 1

ABCA13 "ATP-binding cassette, sub-family A (ABC1), member 13"

BAIAP2 BAI1-associated protein 2

GLB1 "galactosidase, beta 1"

JAZF1 JAZF zinc finger 1

MX2 myxovirus (influenza virus) resistance 2 (mouse)

WDR41 WD repeat domain 41

TMEM63B transmembrane protein 63B

SEMA3D "sema domain, immunoglobulin domain (Ig), short basic domain, secreted, (semaphorin) 3D"

SLC18A2 "solute carrier family 18 (vesicular monoamine), member 2"

ZNF507 zinc finger protein 507

GPAM "glycerol-3-phosphate acyltransferase, mitochondrial"

SIL1 "SIL1 homolog, endoplasmic reticulum chaperone (S. cerevisiae)"

SIN3A "SIN3 homolog A, transcription regulator (yeast)"

PLA2R1 "phospholipase A2 receptor 1, 180kDa"

FANCA "Fanconi anemia, complementation group A"

DIAPH3 diaphanous homolog 3 (Drosophila)

BRP44L brain protein 44-like

VTI1A vesicle transport through interaction with t-SNAREs homolog 1A (yeast)

DYM dymeclin

CEP152 centrosomal protein 152kDa

SNORD22 "small nucleolar RNA, C/D box 22"

BIRC6 baculoviral IAP repeat-containing 6 (apollon)

OS9 -

YEATS2 YEATS domain containing 2

B3GAT1 "beta-1,3-glucuronyltransferase 1 (glucuronosyltransferase P)"

RNF165 ring finger protein 165

DOT1L "DOT1-like, histone H3 methyltransferase (S. cerevisiae)"

RAP1GAP RAP1 GTPase activating protein

KLHL8 kelch-like 8 (Drosophila)

FBXL17 F-box and leucine-rich repeat protein 17

BMP4 bone morphogenetic protein 4

MYH1 "myosin, heavy chain 1, skeletal muscle, adult"

SOX5 SRY (sex determining region Y)-box 5

MAML2 mastermind-like 2 (Drosophila)

TMEM87B transmembrane protein 87B

DENND2C DENN/MADD domain containing 2C

ALPK1 alpha-kinase 1

XYLT2 xylosyltransferase II

DSC1 desmocollin 1

PDE7B phosphodiesterase 7B

ALOX12B "arachidonate 12-lipoxygenase, 12R type"

CCDC67 coiled-coil domain containing 67

ATP5D "ATP synthase, H+ transporting, mitochondrial F1 complex, delta subunit"

EPHB6 EPH receptor B6

RANBP10 RAN binding protein 10

AKAP7 A kinase (PRKA) anchor protein 7

NARS2 asparaginyl-tRNA synthetase 2 (mitochondrial)(putative)

F9 "coagulation factor IX (plasma thromboplastic component, Christmas disease, hemophilia B)"

SYN3 synapsin III

OTUD7A OTU domain containing 7A

MYH7 "myosin, heavy chain 7, cardiac muscle, beta"

ALG2 "asparagine-linked glycosylation 2 homolog (S. cerevisiae, alpha-1,3-mannosyltransferase)"

OTUD1 OTU domain containing 1

FAM83B "family with sequence similarity 83, member B"

FBXO42 F-box protein 42

FANCD2 "Fanconi anemia, complementation group D2"

B4GALT1 "UDP-Gal:betaGlcNAc beta 1,4- galactosyltransferase, polypeptide 1"

TNKS2 "tankyrase, TRF1-interacting ankyrin-related ADP-ribose polymerase 2"

MTA3 "metastasis associated 1 family, member 3"

IBSP "integrin-binding sialoprotein (bone sialoprotein, bone sialoprotein II)"

LGI2 "leucine-rich repeat LGI family, member 2"

AIG1 androgen-induced 1

AGTRAP angiotensin II receptor-associated protein

SORT1 sortilin 1

SLC4A5 "solute carrier family 4, sodium bicarbonate cotransporter, member 5"

RPL18A ribosomal protein L18a

IMPG1 interphotoreceptor matrix proteoglycan 1

MATN1 "matrilin 1, cartilage matrix protein"

IRGQ "immunity-related GTPase family, Q"

DBX1 -

CNTN4 contactin 4

RCC2 regulator of chromosome condensation 2

KRT86 keratin 86

ODF2 outer dense fiber of sperm tails 2

GRM4 "glutamate receptor, metabotropic 4"

GAN giant axonal neuropathy (gigaxonin)

B4GALT4 "UDP-Gal:betaGlcNAc beta 1,4- galactosyltransferase, polypeptide 4"

KHSRP KH-type splicing regulatory protein (FUSE binding protein 2)

MAML3 mastermind-like 3 (Drosophila)

POLR3C polymerase (RNA) III (DNA directed) polypeptide C (62kD)

PABPC3 "poly(A) binding protein, cytoplasmic 3"

CAPN5 calpain 5

SLC14A2 "solute carrier family 14 (urea transporter), member 2"

SLC10A6 "solute carrier family 10 (sodium/bile acid cotransporter family), member 6"

POMP proteasome maturation protein

SYT9 synaptotagmin IX

GNPDA2 glucosamine-6-phosphate deaminase 2

RBP3 "retinol binding protein 3, interstitial"

TNP1 transition protein 1 (during histone to protamine replacement)

ACTBL2 "actin, beta-like 2"

EFHB "EF-hand domain family, member B"

B3GALTL "beta 1,3-galactosyltransferase-like"

MYBPC3 "myosin binding protein C, cardiac"

SMPX "small muscle protein, X-linked"

BDP1 "B double prime 1, subunit of RNA polymerase III transcription initiation factor IIIB"

TMED6 transmembrane emp24 protein transport domain containing 6

TRPC7 "transient receptor potential cation channel, subfamily C, member 7"

PYGO2 pygopus homolog 2 (Drosophila)

SNORA40 "small nucleolar RNA, H/ACA box 40"

SNORA31 "small nucleolar RNA, H/ACA box 31"

SNORA48 "small nucleolar RNA, H/ACA box 48"

SLC4A10 "solute carrier family 4, sodium bicarbonate transporter-like, member 10"

OLAH oleoyl-ACP hydrolase

CACNG2 "calcium channel, voltage-dependent, gamma subunit 2"

TBX4 T-box 4

NPHP4 nephronophthisis 4

NUDT3 nudix (nucleoside diphosphate linked moiety X)-type motif 3

TYW3 tRNA-yW synthesizing protein 3 homolog (S. cerevisiae)

ARSG arylsulfatase G

NMNAT3 nicotinamide nucleotide adenylyltransferase 3

NPBWR1 neuropeptides B/W receptor 1

SLC36A2 "solute carrier family 36 (proton/amino acid symporter), member 2"

ATG7 ATG7 autophagy related 7 homolog (S. cerevisiae)

TPCN2 two pore segment channel 2

ADCK5 aarF domain containing kinase 5

APOBEC3F "apolipoprotein B mRNA editing enzyme, catalytic polypeptide-like 3F"

DDX50 DEAD (Asp-Glu-Ala-Asp) box polypeptide 50

CCDC66 coiled-coil domain containing 66

GPR31 G protein-coupled receptor 31

NFKBIL1 nuclear factor of kappa light polypeptide gene enhancer in B-cells inhibitor-like 1

IQCD IQ motif containing D

ALPI "alkaline phosphatase, intestinal"

MTTP microsomal triglyceride transfer protein

CDH12 "cadherin 12, type 2 (N-cadherin 2)"

HEPHL1 hephaestin-like 1

SERPINA11 "serpin peptidase inhibitor, clade A (alpha-1 antiproteinase, antitrypsin), member 11"

ARMC4 armadillo repeat containing 4

RTDR1 rhabdoid tumor deletion region gene 1

DNAH7 "dynein, axonemal, heavy chain 7"

BRF1 "BRF1 homolog, subunit of RNA polymerase III transcription initiation factor IIIB (S. cerevisiae)"

DBNL drebrin-like

GNG8 "guanine nucleotide binding protein (G protein), gamma 8"

NDST3 N-deacetylase/N-sulfotransferase (heparan glucosaminyl) 3

SLC24A2 "solute carrier family 24 (sodium/potassium/calcium exchanger), member 2"

SPATA16 spermatogenesis associated 16

GALNT9 UDP-N-acetyl-alpha-D-galactosamine:polypeptide N-acetylgalactosaminyltransferase 9 (GalNAc-T9)

XKR5 "XK, Kell blood group complex subunit-related family, member 5"

OTOG otogelin

PCDH15 protocadherin 15

HMGB4 high-mobility group box 4

OTOS otospiralin

GLIPR1L1 GLI pathogenesis-related 1 like 1

SNORA27 "small nucleolar RNA, H/ACA box 27"

SNORA41 "small nucleolar RNA, H/ACA box 41"

SNORA70 "small nucleolar RNA, H/ACA box 70"

SNORD24 "small nucleolar RNA, C/D box 24"

ALS2CR7 "amyotrophic lateral sclerosis 2 (juvenile) chromosome region, candidate 7"

ARL6IP2 ADP-ribosylation factor-like 6 interacting protein 2

CBARA1 calcium binding atopy-related autoantigen 1

CCDC46 coiled-coil domain containing 46

CCDC73 coiled-coil domain containing 73

CDC2L6 cell division cycle 2-like 6 (CDK8-like)

CENTG2 "centaurin, gamma 2"

FREQ frequenin homolog (Drosophila)

LARP5 "La ribonucleoprotein domain family, member 5"

LASS3 "LAG1 homolog, ceramide synthase 3 (S. cerevisiae)"

LRRC30 leucine rich repeat containing 30

M6PRBP1 mannose-6-phosphate receptor binding protein 1

MAGEB18 "melanoma antigen family B, 18"

MAP3K7IP3 mitogen-activated protein kinase kinase kinase 7 interacting protein 3

MOBKL2B "MOB1, Mps One Binder kinase activator-like 2B (yeast)"

NA neurocanthocytosis

NEK5 NIMA (never in mitosis gene a)-related kinase 5

PFTK1 PFTAIRE protein kinase 1

PHGDHL1 phosphoglycerate dehydrogenase like 1

SFRS14 "splicing factor, arginine/serine-rich 14"

SFRS8 "splicing factor, arginine/serine-rich 8 (suppressor-of-white-apricot homolog, Drosophila)"

SNORA17 "small nucleolar RNA, H/ACA box 17"

SNORA52 "small nucleolar RNA, H/ACA box 52"

WDR51B WD repeat domain 51B

ZNF291 zinc finger protein 291

C3

MLLT7 MLLT7

overlap matrix by gene and geneset TTGTTT_V$FOXO4_01 [2061] V$FOXO4_01 [243] "AAAGACA,MIR-511 [202]" "TAGCTTT,MIR-9 [236]" V$FOXJ2_02 [237] TGTTTGY_V$HNF3_Q6 [738] AACTTT_UNKNOWN [1890] V$HTF_01 [72] V$PXR_Q2 [256] V$SRY_01 [224] description

BCL11B X X X X X X B-cell CLL/lymphoma 11B (zinc finger protein)

STX16 X X X syntaxin 16

ERG X X X X X v-ets erythroblastosis virus E26 oncogene homolog (avian)

PDE4D X X X X X "phosphodiesterase 4D, cAMP-specific (phosphodiesterase E3 dunce homolog, Drosophila)"

NRG1 X X X X X neuregulin 1

CACNG2 X X X X "calcium channel, voltage-dependent, gamma subunit 2"

NMNAT2 X X X X X nicotinamide nucleotide adenylyltransferase 2

PDCD4 X X X X programmed cell death 4 (neoplastic transformation inhibitor)

SKAP1 X X X src kinase associated phosphoprotein 1

FRMD4A X X X FERM domain containing 4A

H2AFV X X X "H2A histone family, member V"

OLIG3 X X X X oligodendrocyte transcription factor 3

RUNX1 X X X runt-related transcription factor 1 (acute myeloid leukemia 1; aml1 oncogene)

MAML3 X X X mastermind-like 3 (Drosophila)

SLC25A28 X X X "solute carrier family 25, member 28"

VLDLR X X X very low density lipoprotein receptor

TBX4 X X T-box 4

KCNJ15 X X "potassium inwardly-rectifying channel, subfamily J, member 15"

DST X X dystonin

RANBP10 X X RAN binding protein 10

SLC4A4 X X "solute carrier family 4, sodium bicarbonate cotransporter, member 4"

SLC36A2 X X "solute carrier family 36 (proton/amino acid symporter), member 2"

KRT86 X X keratin 86

HAS2 X X X hyaluronan synthase 2

BTG1 X X X X "B-cell translocation gene 1, anti-proliferative"

DUSP6 X X X X dual specificity phosphatase 6

CEP350 X X X centrosomal protein 350kDa

PCDH17 X X protocadherin 17

NTRK2 X X "neurotrophic tyrosine kinase, receptor, type 2"

TEAD1 X X X X X X TEA domain family member 1 (SV40 transcriptional enhancer factor)

SMARCA2 X X X X "SWI/SNF related, matrix associated, actin dependent regulator of chromatin, subfamily a, member 2"

ATXN1 X X X X ataxin 1

SFMBT1 X X Scm-like with four mbt domains 1

PIGF X X "phosphatidylinositol glycan anchor biosynthesis, class F"

CDH10 X X X X X "cadherin 10, type 2 (T2-cadherin)"

FOXP1 X X X forkhead box P1

SYT6 X X X synaptotagmin VI

SATB1 X X X special AT-rich sequence binding protein 1 (binds to nuclear matrix/scaffold-associating DNA's)

PRDM12 X X X PR domain containing 12

GRIN2B X X X X "glutamate receptor, ionotropic, N-methyl D-aspartate 2B"

FAM105A X X "family with sequence similarity 105, member A"

RFX4 X X X X X "regulatory factor X, 4 (influences HLA class II expression)"

MEIS1 X X X X "Meis1, myeloid ecotropic viral integration site 1 homolog (mouse)"

SOX5 X X X X SRY (sex determining region Y)-box 5

NAV3 X X X X neuron navigator 3

NTN1 X X X netrin 1

MAPK10 X X X mitogen-activated protein kinase 10

GRID2 X X X "glutamate receptor, ionotropic, delta 2"

SLC6A1 X X X "solute carrier family 6 (neurotransmitter transporter, GABA), member 1"

RGS3 X X X regulator of G-protein signalling 3

EML1 X X X echinoderm microtubule associated protein like 1

RBP3 X X X "retinol binding protein 3, interstitial"

SLC38A4 X X X "solute carrier family 38, member 4"

SRPK2 X X SFRS protein kinase 2

OGT X X O-linked N-acetylglucosamine (GlcNAc) transferase (UDP-N-acetylglucosamine:polypeptide-N-acetylglucosaminyl transferase)

SDK2 X X sidekick homolog 2 (chicken)

NFE2L3 X X nuclear factor (erythroid-derived 2)-like 3

HTR1B X X 5-hydroxytryptamine (serotonin) receptor 1B

MUM1L1 X X melanoma associated antigen (mutated) 1-like 1

TMED10 X X transmembrane emp24-like trafficking protein 10 (yeast)

CDH12 X X "cadherin 12, type 2 (N-cadherin 2)"

PRRX1 X X X X X paired related homeobox 1

MBNL2 X X X muscleblind-like 2 (Drosophila)

GRIK3 X X X "glutamate receptor, ionotropic, kainate 3"

SMAD1 X X X "SMAD, mothers against DPP homolog 1 (Drosophila)"

NR3C2 X X X "nuclear receptor subfamily 3, group C, member 2"

SYTL2 X X X synaptotagmin-like 2

ANK3 X X X "ankyrin 3, node of Ranvier (ankyrin G)"

CACNA1E X X X "calcium channel, voltage-dependent, alpha 1E subunit"

STARD13 X X START domain containing 13

MAP2K6 X X mitogen-activated protein kinase kinase 6

EHBP1 X X EH domain binding protein 1

SLC24A2 X X "solute carrier family 24 (sodium/potassium/calcium exchanger), member 2"

PCDH7 X X BH-protocadherin (brain-heart)

LRRC3B X X leucine rich repeat containing 3B

SNTB2 X X "syntrophin, beta 2 (dystrophin-associated protein A1, 59kDa, basic component 2)"

C1QTNF7 X X C1q and tumor necrosis factor related protein 7

STC2 X X stanniocalcin 2

BIRC6 X X baculoviral IAP repeat-containing 6 (apollon)

ZBTB20 X X zinc finger and BTB domain containing 20

CALN1 X X calneuron 1

MLLT3 X X "myeloid/lymphoid or mixed-lineage leukemia (trithorax homolog, Drosophila); translocated to, 3"

MID2 X X midline 2

GPR22 X X G protein-coupled receptor 22

LAMA4 X X "laminin, alpha 4"

PREP X X prolyl endopeptidase

SLC18A2 X X "solute carrier family 18 (vesicular monoamine), member 2"

ARID4B X X AT rich interactive domain 4B (RBP1- like)

MAML2 X X mastermind-like 2 (Drosophila)

TNKS2 X X "tankyrase, TRF1-interacting ankyrin-related ADP-ribose polymerase 2"

SORT1 X X sortilin 1

ABLIM1 X X actin binding LIM protein 1

DPYSL4 X X dihydropyrimidinase-like 4

SH3GLB1 X X SH3-domain GRB2-like endophilin B1

DLC1 X deleted in liver cancer 1

SUMO1 X SMT3 suppressor of mif two 3 homolog 1 (S. cerevisiae)

BMP4 X bone morphogenetic protein 4

TIAM1 X T-cell lymphoma invasion and metastasis 1

TSPAN5 X tetraspanin 5

IBSP X "integrin-binding sialoprotein (bone sialoprotein, bone sialoprotein II)"

DDIT4L X DNA-damage-inducible transcript 4-like

CTLA4 X cytotoxic T-lymphocyte-associated protein 4

BTG3 X "BTG family, member 3"

ETS2 X v-ets erythroblastosis virus E26 oncogene homolog 2 (avian)

KLHL13 X kelch-like 13 (Drosophila)

PRKD1 X protein kinase D1

EPHB6 X EPH receptor B6

DENND2C X DENN/MADD domain containing 2C

SIRPA X signal-regulatory protein alpha

FRMPD1 X FERM and PDZ domain containing 1

ST6GAL1 X "ST6 beta-galactosamide alpha-2,6-sialyltranferase 1"

CCDC67 X coiled-coil domain containing 67

DCUN1D4 X "DCN1, defective in cullin neddylation 1, domain containing 4 (S. cerevisiae)"

PRDM8 X PR domain containing 8

LRRN3 X leucine rich repeat neuronal 3

FAIM2 X Fas apoptotic inhibitory molecule 2

NUDT3 X nudix (nucleoside diphosphate linked moiety X)-type motif 3

PPM1L X protein phosphatase 1 (formerly 2C)-like

MPZL1 X myelin protein zero-like 1

CD24 X CD24 molecule

PAWR X "PRKC, apoptosis, WT1, regulator"

MYH7 X "myosin, heavy chain 7, cardiac muscle, beta"

DNAH7 X "dynein, axonemal, heavy chain 7"

GNE X glucosamine (UDP-N-acetyl)-2-epimerase/N-acetylmannosamine kinase

UGT8 X UDP glycosyltransferase 8 (UDP-galactose ceramide galactosyltransferase)

EPHB1 X X EPH receptor B1

TRIM2 X X X tripartite motif-containing 2

DSC1 X X desmocollin 1

ARHGEF17 X X Rho guanine nucleotide exchange factor (GEF) 17

SEMA3F X X "sema domain, immunoglobulin domain (Ig), short basic domain, secreted, (semaphorin) 3F"

EYA4 X X eyes absent homolog 4 (Drosophila)

CCND1 X X cyclin D1

SYT11 X X synaptotagmin XI

SLC6A6 X X "solute carrier family 6 (neurotransmitter transporter, taurine), member 6"

CAMTA1 X calmodulin binding transcription activator 1

RGL1 X ral guanine nucleotide dissociation stimulator-like 1

ROBO2 X "roundabout, axon guidance receptor, homolog 2 (Drosophila)"

VAV3 X vav 3 oncogene

CLK2 X CDC-like kinase 2

ENPP1 X ectonucleotide pyrophosphatase/phosphodiesterase 1

ZFHX4 X X X zinc finger homeodomain 4

HIPK1 X X homeodomain interacting protein kinase 1

JAZF1 X X JAZF zinc finger 1

CNTN4 X X contactin 4

BACH2 X "BTB and CNC homology 1, basic leucine zipper transcription factor 2"

SORCS1 X sortilin-related VPS10 domain containing receptor 1

GABBR2 X "gamma-aminobutyric acid (GABA) B receptor, 2"

ATP11A X "ATPase, Class VI, type 11A"

CCND2 X cyclin D2

VPS13A X vacuolar protein sorting 13 homolog A (S. cerevisiae)

E2F7 X E2F transcription factor 7

PRKCE X "protein kinase C, epsilon"

SKAP2 X src kinase associated phosphoprotein 2

MAP3K7 X mitogen-activated protein kinase kinase kinase 7

PDK1 X "pyruvate dehydrogenase kinase, isozyme 1"

LTBP1 X X X latent transforming growth factor beta binding protein 1

ATP2B4 X X "ATPase, Ca++ transporting, plasma membrane 4"

B3GALTL X X "beta 1,3-galactosyltransferase-like"

PYGO2 X X pygopus homolog 2 (Drosophila)

ATRNL1 X X X attractin-like 1

CD180 X CD180 molecule

UTRN X utrophin (homologous to dystrophin)

XPOT X "exportin, tRNA (nuclear export receptor for tRNAs)"

ARSG X arylsulfatase G

TAOK3 X X X TAO kinase 3

RFX3 X X "regulatory factor X, 3 (influences HLA class II expression)"

CNTNAP2 X X contactin associated protein-like 2

IRX5 X X iroquois homeobox protein 5

SYNE2 X X "spectrin repeat containing, nuclear envelope 2"

EFNA5 X X ephrin-A5

RERE X arginine-glutamic acid dipeptide (RE) repeats

TRIM44 X tripartite motif-containing 44

F9 X "coagulation factor IX (plasma thromboplastic component, Christmas disease, hemophilia B)"

LGI2 X "leucine-rich repeat LGI family, member 2"

PTGER2 X "prostaglandin E receptor 2 (subtype EP2), 53kDa"

MN1 X X meningioma (disrupted in balanced translocation) 1

FAM5C X X "family with sequence similarity 5, member C"

HCN1 X X hyperpolarization activated cyclic nucleotide-gated potassium channel 1

CACNB3 X X "calcium channel, voltage-dependent, beta 3 subunit"

DHX40 X DEAH (Asp-Glu-Ala-His) box polypeptide 40

SOCS1 X suppressor of cytokine signaling 1

SLC4A10 X "solute carrier family 4, sodium bicarbonate transporter-like, member 10"

SETD2 X SET domain containing 2

ITSN1 X intersectin 1 (SH3 domain protein)

ELOVL5 X "ELOVL family member 5, elongation of long chain fatty acids (FEN1/Elo2, SUR4/Elo3-like, yeast)"

XYLT2 X xylosyltransferase II

SMPX X "small muscle protein, X-linked"

NPHP4 X nephronophthisis 4

PPARD X "peroxisome proliferative activated receptor, delta"

STK39 X "serine threonine kinase 39 (STE20/SPS1 homolog, yeast)"

MYH10 X "myosin, heavy chain 10, non-muscle"

NDST3 X N-deacetylase/N-sulfotransferase (heparan glucosaminyl) 3

DGKI X "diacylglycerol kinase, iota"

LARGE X like-glycosyltransferase

SREBF2 X sterol regulatory element binding transcription factor 2

PTK2 X PTK2 protein tyrosine kinase 2

DCAKD X dephospho-CoA kinase domain containing

MYH1 X "myosin, heavy chain 1, skeletal muscle, adult"

SYT9 X synaptotagmin IX

TNFSF10 X "tumor necrosis factor (ligand) superfamily, member 10"

TRAF3 X TNF receptor-associated factor 3

ACSBG1 X acyl-CoA synthetase bubblegum family member 1

KCNIP1 X Kv channel interacting protein 1

LDLRAD3 X low density lipoprotein receptor class A domain containing 3

PIAS2 X "protein inhibitor of activated STAT, 2"

ARMC4 X armadillo repeat containing 4

PTPRC X "protein tyrosine phosphatase, receptor type, C"

SEMA4A X "sema domain, immunoglobulin domain (Ig), transmembrane domain (TM) and short cytoplasmic domain, (semaphorin) 4A"

ARHGAP18 X Rho GTPase activating protein 18

IMPG1 X interphotoreceptor matrix proteoglycan 1

EGLN1 egl nine homolog 1 (C. elegans)

SIN3A "SIN3 homolog A, transcription regulator (yeast)"

PRKCH "protein kinase C, eta"

FYN "FYN oncogene related to SRC, FGR, YES"

EFHB "EF-hand domain family, member B"

CD109 CD109 molecule

MTTP microsomal triglyceride transfer protein

SYT10 synaptotagmin X

MAP3K8 mitogen-activated protein kinase kinase kinase 8

SYT1 synaptotagmin I

SERTAD2 SERTA domain containing 2

TBC1D15 "TBC1 domain family, member 15"

PTPN2 "protein tyrosine phosphatase, non-receptor type 2"

PDZRN3 PDZ domain containing RING finger 3

LRBA "LPS-responsive vesicle trafficking, beach and anchor containing"

USP47 ubiquitin specific peptidase 47

FMN2 formin 2

TPST1 tyrosylprotein sulfotransferase 1

CASP8AP2 CASP8 associated protein 2

ARID2 "AT rich interactive domain 2 (ARID, RFX-like)"

PDE7B phosphodiesterase 7B

CSMD2 CUB and Sushi multiple domains 2

GATA3 GATA binding protein 3

MKX mohawk homeobox

HSPA4 heat shock 70kDa protein 4

FBXO42 F-box protein 42

RNF165 ring finger protein 165

PSD3 pleckstrin and Sec7 domain containing 3

AP3B1 "adaptor-related protein complex 3, beta 1 subunit"

WDFY3 WD repeat and FYVE domain containing 3

CHD9 chromodomain helicase DNA binding protein 9

AKAP7 A kinase (PRKA) anchor protein 7

PPP2R5C "protein phosphatase 2, regulatory subunit B (B56), gamma isoform"

SEC23A Sec23 homolog A (S. cerevisiae)

COPS8 COP9 constitutive photomorphogenic homolog subunit 8 (Arabidopsis)

INHBB "inhibin, beta B (activin AB beta polypeptide)"

TTYH3 tweety homolog 3 (Drosophila)

B4GALT5 "UDP-Gal:betaGlcNAc beta 1,4- galactosyltransferase, polypeptide 5"

TMEM63B transmembrane protein 63B

CCRN4L CCR4 carbon catabolite repression 4-like (S. cerevisiae)

EDARADD EDAR-associated death domain

ATP10A "ATPase, Class V, type 10A"

CTDSPL "CTD (carboxy-terminal domain, RNA polymerase II, polypeptide A) small phosphatase-like"

PRR16 proline rich 16

MGAT5B "mannosyl (alpha-1,6-)-glycoprotein beta-1,6-N-acetyl-glucosaminyltransferase, isozyme B"

ASPH aspartate beta-hydroxylase

ABHD6 abhydrolase domain containing 6

BRP44L brain protein 44-like

FBXL17 F-box and leucine-rich repeat protein 17

GAN giant axonal neuropathy (gigaxonin)

DPP4 "dipeptidyl-peptidase 4 (CD26, adenosine deaminase complexing protein 2)"

TOPORS "topoisomerase I binding, arginine/serine-rich"

SYN2 synapsin II

DDHD1 DDHD domain containing 1

TANC1 "tetratricopeptide repeat, ankyrin repeat and coiled-coil containing 1"

DOCK1 dedicator of cytokinesis 1

IGSF11 "immunoglobulin superfamily, member 11"

SMAD4 "SMAD, mothers against DPP homolog 4 (Drosophila)"

EDN3 endothelin 3

ACTR10 actin-related protein 10 homolog (S. cerevisiae)

USP48 ubiquitin specific peptidase 48

DYNLL2 "dynein, light chain, LC8-type 2"

DIP2A DIP2 disco-interacting protein 2 homolog A (Drosophila)

SNX2 sorting nexin 2

CDK8 cyclin-dependent kinase 8

B4GALT1 "UDP-Gal:betaGlcNAc beta 1,4- galactosyltransferase, polypeptide 1"

ARHGAP26 Rho GTPase activating protein 26

NMNAT3 nicotinamide nucleotide adenylyltransferase 3

TMEM117 transmembrane protein 117

ABI1 abl-interactor 1

SYN3 synapsin III

BAIAP2 BAI1-associated protein 2

ERC1 ELKS/RAB6-interacting/CAST family member 1

NARS2 asparaginyl-tRNA synthetase 2 (mitochondrial)(putative)

PPHLN1 periphilin 1

GPAM "glycerol-3-phosphate acyltransferase, mitochondrial"

DPY19L3 dpy-19-like 3 (C. elegans)

SLCO5A1 "solute carrier organic anion transporter family, member 5A1"

B3GAT1 "beta-1,3-glucuronyltransferase 1 (glucuronosyltransferase P)"

KCNA6 "potassium voltage-gated channel, shaker-related subfamily, member 6"

YEATS2 YEATS domain containing 2

TBC1D23 "TBC1 domain family, member 23"

RIC8B resistance to inhibitors of cholinesterase 8 homolog B (C. elegans)

FGD5 "FYVE, RhoGEF and PH domain containing 5"

KHSRP KH-type splicing regulatory protein (FUSE binding protein 2)

PJA2 "praja 2, RING-H2 motif containing"

ERGIC1 endoplasmic reticulum-golgi intermediate compartment (ERGIC) 1

PHACTR1 phosphatase and actin regulator 1

SIM2 single-minded homolog 2 (Drosophila)

ZNF704 zinc finger protein 704

FBN1 fibrillin 1

RSU1 Ras suppressor protein 1

OSBP2 oxysterol binding protein 2

COTL1 coactosin-like 1 (Dictyostelium)

RCC2 regulator of chromosome condensation 2

CDC14A CDC14 cell division cycle 14 homolog A (S. cerevisiae)

PEPD peptidase D

ATP13A3 ATPase type 13A3

PLEKHA5 "pleckstrin homology domain containing, family A member 5"

UBE2V2 ubiquitin-conjugating enzyme E2 variant 2

ZNF507 zinc finger protein 507

ZNF536 zinc finger protein 536

PTF1A "pancreas specific transcription factor, 1a"

MKLN1 "muskelin 1, intracellular mediator containing kelch motifs"

SPSB1 splA/ryanodine receptor domain and SOCS box containing 1

TBC1D22A "TBC1 domain family, member 22A"

GABBR1 "gamma-aminobutyric acid (GABA) B receptor, 1"

USP9X "ubiquitin specific peptidase 9, X-linked"

FBXL2 F-box and leucine-rich repeat protein 2

LRRFIP1 leucine rich repeat (in FLII) interacting protein 1

ALG2 "asparagine-linked glycosylation 2 homolog (S. cerevisiae, alpha-1,3-mannosyltransferase)"

GNPDA2 glucosamine-6-phosphate deaminase 2

NEK7 NIMA (never in mitosis gene a)-related kinase 7

SORCS2 sortilin-related VPS10 domain containing receptor 2

AIG1 androgen-induced 1

OTOS otospiralin

IDE insulin-degrading enzyme

MYO7A myosin VIIA

ACLY ATP citrate lyase

TMEM67 transmembrane protein 67

RASA2 RAS p21 protein activator 2

ETNK1 ethanolamine kinase 1

CYYR1 cysteine/tyrosine-rich 1

ARID5B AT rich interactive domain 5B (MRF1-like)

SPOCK1 "sparc/osteonectin, cwcv and kazal-like domains proteoglycan (testican) 1"

ALPK1 alpha-kinase 1

PI4K2B phosphatidylinositol 4-kinase type 2 beta

DBNL drebrin-like

HMGB4 high-mobility group box 4

FGFRL1 fibroblast growth factor receptor-like 1

MATN3 matrilin 3

GNG8 "guanine nucleotide binding protein (G protein), gamma 8"

NFATC2 "nuclear factor of activated T-cells, cytoplasmic, calcineurin-dependent 2"

SETBP1 SET binding protein 1

LSM6 "LSM6 homolog, U6 small nuclear RNA associated (S. cerevisiae)"

MATN1 "matrilin 1, cartilage matrix protein"

FBXL14 F-box and leucine-rich repeat protein 14

RIN3 Ras and Rab interactor 3

ECEL1 endothelin converting enzyme-like 1

ARHGAP22 Rho GTPase activating protein 22

SEC23B Sec23 homolog B (S. cerevisiae)

LAPTM4A lysosomal-associated protein transmembrane 4 alpha

VDAC1 voltage-dependent anion channel 1

PABPC3 "poly(A) binding protein, cytoplasmic 3"

DOK6 docking protein 6

NCOR1 nuclear receptor co-repressor 1

FGF21 fibroblast growth factor 21

METTL8 methyltransferase like 8

BET1 BET1 homolog (S. cerevisiae)

PSMD1 "proteasome (prosome, macropain) 26S subunit, non-ATPase, 1"

EIF2B3 "eukaryotic translation initiation factor 2B, subunit 3 gamma, 58kDa"

PRMT2 protein arginine methyltransferase 2

DCC deleted in colorectal carcinoma

RRS1 RRS1 ribosome biogenesis regulator homolog (S. cerevisiae)

DNAJC3 "DnaJ (Hsp40) homolog, subfamily C, member 3"

POLR3C polymerase (RNA) III (DNA directed) polypeptide C (62kD)

FANCA "Fanconi anemia, complementation group A"

SLC4A5 "solute carrier family 4, sodium bicarbonate cotransporter, member 5"

CEP135 centrosomal protein 135kDa

NHLRC2 NHL repeat containing 2

COL4A4 "collagen, type IV, alpha 4"

MYBPC3 "myosin binding protein C, cardiac"

LHFPL2 lipoma HMGIC fusion partner-like 2

CHRM3 "cholinergic receptor, muscarinic 3"

TBC1D5 "TBC1 domain family, member 5"

LTBP2 latent transforming growth factor beta binding protein 2

DLGAP1 "discs, large (Drosophila) homolog-associated protein 1"

VTI1A vesicle transport through interaction with t-SNAREs homolog 1A (yeast)

ODF2 outer dense fiber of sperm tails 2

NDUFB3 "NADH dehydrogenase (ubiquinone) 1 beta subcomplex, 3, 12kDa"

PTPN14 "protein tyrosine phosphatase, non-receptor type 14"

ANKRD55 ankyrin repeat domain 55

ANKH "ankylosis, progressive homolog (mouse)"

ZCCHC9 "zinc finger, CCHC domain containing 9"

TPCN2 two pore segment channel 2

MTR 5-methyltetrahydrofolate-homocysteine methyltransferase

GNG10 "guanine nucleotide binding protein (G protein), gamma 10"

COL5A1 "collagen, type V, alpha 1"

GALNT9 UDP-N-acetyl-alpha-D-galactosamine:polypeptide N-acetylgalactosaminyltransferase 9 (GalNAc-T9)

CACNA1B "calcium channel, voltage-dependent, L type, alpha 1B subunit"

DOT1L "DOT1-like, histone H3 methyltransferase (S. cerevisiae)"

TNP1 transition protein 1 (during histone to protamine replacement)

DDX50 DEAD (Asp-Glu-Ala-Asp) box polypeptide 50

ADK adenosine kinase

ACTR2 ARP2 actin-related protein 2 homolog (yeast)

TTLL7 "tubulin tyrosine ligase-like family, member 7"

CAPN5 calpain 5

OS9 -

CCNF cyclin F

PLA2R1 "phospholipase A2 receptor 1, 180kDa"

POLR1B "polymerase (RNA) I polypeptide B, 128kDa"

VDAC3 voltage-dependent anion channel 3

CTDP1 "CTD (carboxy-terminal domain, RNA polymerase II, polypeptide A) phosphatase, subunit 1"

PIGT "phosphatidylinositol glycan anchor biosynthesis, class T"

AGTRAP angiotensin II receptor-associated protein

BCAM basal cell adhesion molecule (Lutheran blood group)

AKAP9 A kinase (PRKA) anchor protein (yotiao) 9

FANCD2 "Fanconi anemia, complementation group D2"

SCNN1G "sodium channel, nonvoltage-gated 1, gamma"

SHROOM3 shroom family member 3

TTLL5 "tubulin tyrosine ligase-like family, member 5"

TBX21 T-box 21

DIAPH3 diaphanous homolog 3 (Drosophila)

CSF3R colony stimulating factor 3 receptor (granulocyte)

SLC37A1 "solute carrier family 37 (glycerol-3-phosphate transporter), member 1"

AHDC1 "AT hook, DNA binding motif, containing 1"

IQCD IQ motif containing D

PDIA5 "protein disulfide isomerase family A, member 5"

FBLN1 fibulin 1

GLRX2 glutaredoxin 2

NPBWR1 neuropeptides B/W receptor 1

BDP1 "B double prime 1, subunit of RNA polymerase III transcription initiation factor IIIB"

RBM10 RNA binding motif protein 10

SNRPB small nuclear ribonucleoprotein polypeptides B and B1

OPRD1 "opioid receptor, delta 1"

ALOX12B "arachidonate 12-lipoxygenase, 12R type"

CEACAM1 carcinoembryonic antigen-related cell adhesion molecule 1 (biliary glycoprotein)

GSR glutathione reductase

KCNAB2 "potassium voltage-gated channel, shaker-related subfamily, beta member 2"

RPL18A ribosomal protein L18a

PCDH15 protocadherin 15

CYP19A1 "cytochrome P450, family 19, subfamily A, polypeptide 1"

TMED6 transmembrane emp24 protein transport domain containing 6

AIM1 absent in melanoma 1

GRM4 "glutamate receptor, metabotropic 4"

CCDC73 coiled-coil domain containing 73

ABCC8 "ATP-binding cassette, sub-family C (CFTR/MRP), member 8"

NFKBIL1 nuclear factor of kappa light polypeptide gene enhancer in B-cells inhibitor-like 1

NDUFA8 "NADH dehydrogenase (ubiquinone) 1 alpha subcomplex, 8, 19kDa"

NSMCE2 "non-SMC element 2 homolog (MMS21, S. cerevisiae)"

ALPI "alkaline phosphatase, intestinal"

CAMP cathelicidin antimicrobial peptide

ATP7B "ATPase, Cu++ transporting, beta polypeptide"

PHKB "phosphorylase kinase, beta"

GPR83 G protein-coupled receptor 83

DYM dymeclin

WDHD1 WD repeat and HMG-box DNA binding protein 1

TPK1 thiamin pyrophosphokinase 1

POMP proteasome maturation protein

SEMA3D "sema domain, immunoglobulin domain (Ig), short basic domain, secreted, (semaphorin) 3D"

TMEM87B transmembrane protein 87B

LRRC28 leucine rich repeat containing 28

COX6A1 cytochrome c oxidase subunit VIa polypeptide 1

STRAP serine/threonine kinase receptor associated protein

CYBASC3 "cytochrome b, ascorbate dependent 3"

TTC14 tetratricopeptide repeat domain 14

SPATA16 spermatogenesis associated 16

ATP5D "ATP synthase, H+ transporting, mitochondrial F1 complex, delta subunit"

ADCK5 aarF domain containing kinase 5

SLCO4A1 "solute carrier organic anion transporter family, member 4A1"

SNRPE small nuclear ribonucleoprotein polypeptide E

RTDR1 rhabdoid tumor deletion region gene 1

GPR31 G protein-coupled receptor 31

TRPC7 "transient receptor potential cation channel, subfamily C, member 7"

MLPH melanophilin

ABCA13 "ATP-binding cassette, sub-family A (ABC1), member 13"

ACADM "acyl-Coenzyme A dehydrogenase, C-4 to C-12 straight chain"

ACTBL2 "actin, beta-like 2"

ADH5 "alcohol dehydrogenase 5 (class III), chi polypeptide"

ALS2CR7 "amyotrophic lateral sclerosis 2 (juvenile) chromosome region, candidate 7"

APOBEC3F "apolipoprotein B mRNA editing enzyme, catalytic polypeptide-like 3F"

ARL6IP2 ADP-ribosylation factor-like 6 interacting protein 2

ATG10 ATG10 autophagy related 10 homolog (S. cerevisiae)

ATG7 ATG7 autophagy related 7 homolog (S. cerevisiae)

B4GALT4 "UDP-Gal:betaGlcNAc beta 1,4- galactosyltransferase, polypeptide 4"

BRF1 "BRF1 homolog, subunit of RNA polymerase III transcription initiation factor IIIB (S. cerevisiae)"

CABIN1 -

CBARA1 calcium binding atopy-related autoantigen 1

CCDC46 coiled-coil domain containing 46

CCDC66 coiled-coil domain containing 66

CD302 CD302 molecule

CDC2L6 cell division cycle 2-like 6 (CDK8-like)

CENTG2 "centaurin, gamma 2"

CEP152 centrosomal protein 152kDa

CROCC "ciliary rootlet coiled-coil, rootletin"

DBX1 -

ELTD1 "EGF, latrophilin and seven transmembrane domain containing 1"

FAM83B "family with sequence similarity 83, member B"

FREM2 FRAS1 related extracellular matrix protein 2

FREQ frequenin homolog (Drosophila)

FTO -

GLB1 "galactosidase, beta 1"

GLIPR1L1 GLI pathogenesis-related 1 like 1

HEPHL1 hephaestin-like 1

INCENP inner centromere protein antigens 135/155kDa

IRGM "immunity-related GTPase family, M"

IRGQ "immunity-related GTPase family, Q"

KLHL8 kelch-like 8 (Drosophila)

LARP5 "La ribonucleoprotein domain family, member 5"

LASS3 "LAG1 homolog, ceramide synthase 3 (S. cerevisiae)"

LRRC30 leucine rich repeat containing 30

M6PRBP1 mannose-6-phosphate receptor binding protein 1

MAGEB18 "melanoma antigen family B, 18"

MAP3K7IP3 mitogen-activated protein kinase kinase kinase 7 interacting protein 3

METTL7A methyltransferase like 7A

MOBKL2B "MOB1, Mps One Binder kinase activator-like 2B (yeast)"

MTA3 "metastasis associated 1 family, member 3"

MX2 myxovirus (influenza virus) resistance 2 (mouse)

NA neurocanthocytosis

NEK5 NIMA (never in mitosis gene a)-related kinase 5

NSMCE1 non-SMC element 1 homolog (S. cerevisiae)

ODZ2 "odz, odd Oz/ten-m homolog 2 (Drosophila)"

ODZ3 "odz, odd Oz/ten-m homolog 3 (Drosophila)"

OLAH oleoyl-ACP hydrolase

OTOG otogelin

OTUD1 OTU domain containing 1

OTUD7A OTU domain containing 7A

PFTK1 PFTAIRE protein kinase 1

PHGDHL1 phosphoglycerate dehydrogenase like 1

PPP1R1C "protein phosphatase 1, regulatory (inhibitor) subunit 1C"

PRKAR1B "protein kinase, cAMP-dependent, regulatory, type I, beta"

QDPR quinoid dihydropteridine reductase

RAP1GAP RAP1 GTPase activating protein

S100A11 S100 calcium binding protein A11

SCP2 sterol carrier protein 2

SERPINA11 "serpin peptidase inhibitor, clade A (alpha-1 antiproteinase, antitrypsin), member 11"

SETD7 SET domain containing (lysine methyltransferase) 7

SFRS14 "splicing factor, arginine/serine-rich 14"

SFRS8 "splicing factor, arginine/serine-rich 8 (suppressor-of-white-apricot homolog, Drosophila)"

SHE Src homology 2 domain containing E

SIL1 "SIL1 homolog, endoplasmic reticulum chaperone (S. cerevisiae)"

SLC10A6 "solute carrier family 10 (sodium/bile acid cotransporter family), member 6"

SLC14A2 "solute carrier family 14 (urea transporter), member 2"

SLC30A10 "solute carrier family 30, member 10"

SMYD3 SET and MYND domain containing 3

SNORA17 "small nucleolar RNA, H/ACA box 17"

SNORA27 "small nucleolar RNA, H/ACA box 27"

SNORA31 "small nucleolar RNA, H/ACA box 31"

SNORA40 "small nucleolar RNA, H/ACA box 40"

SNORA41 "small nucleolar RNA, H/ACA box 41"

SNORA48 "small nucleolar RNA, H/ACA box 48"

SNORA52 "small nucleolar RNA, H/ACA box 52"

SNORA70 "small nucleolar RNA, H/ACA box 70"

SNORD22 "small nucleolar RNA, C/D box 22"

SNORD24 "small nucleolar RNA, C/D box 24"

SYK spleen tyrosine kinase

TFPI tissue factor pathway inhibitor (lipoprotein-associated coagulation inhibitor)

TYW3 tRNA-yW synthesizing protein 3 homolog (S. cerevisiae)

UCP1 "uncoupling protein 1 (mitochondrial, proton carrier)"

WDR41 WD repeat domain 41

WDR51B WD repeat domain 51B

XKR5 "XK, Kell blood group complex subunit-related family, member 5"

XPA "xeroderma pigmentosum, complementation group A"

ZNF291 zinc finger protein 291 MBD- Differentially Methylated region-neighboring genes (nearest)

"chemical and genetic perturbation analysis, C2-CGP"

"motif gene sets analysis , C3"

Gene Set Name [# Genes (K)] Description # Genes in Overlap (k) p value

DACOSTA_UV_RESPONSE_VIA_ERCC3_DN [855] Genes down-regulated in fibroblasts expressing mutant forms of ERCC3 [GeneID=2071] after UV irradiation. 66 1.25 e-14

DACOSTA_UV_RESPONSE_VIA_ERCC3_COMMON_DN_DN [483] "Common down-regulated transcripts in fibroblasts expressing either XP/CS or TDD mutant forms of ERCC3 [GeneID=2071], after UVC irradiation." 37 1.34 e-8

ONDER_CDH1_TARGETS_2_UP [256] Genes up-regulated in HMLE cells (immortalized nontransformed mammary epithelium) after E-cadhedrin (CDH1) [GeneID=999] knockdown by RNAi. 25 3.2 e-8

CUI_TCF21_TARGETS_2_DN [830] All significantly down-regulated genes in kidney glomeruli isolated from TCF21 [Gene ID=6943] knockout mice. 49 2.51 e-7

IKEDA_MIR30_TARGETS_UP [116] Genes up-regulated in hypertrophic hearts (due to expression of constitutively active form of PPP3CA [GeneID=5530]) and predicted to be targets of miR-30 microRNA. 15 5.36 e-7

ZHENG_BOUND_BY_FOXP3 [491] Genes whose promoters are bound by FOXP3 [GeneID=50943] based an a ChIP-chip analysis. 34 5.66 e-7

GOBERT_OLIGODENDROCYTE_DIFFERENTIATIONION_DN [1080] Genes down-regulated during differentiation of Oli-Neu cells (oligodendroglial precursor) in response to PD174265 [PubChemID=4709]. 57 1 e-6

MEISSNER_BRAIN_HCP_WITH_H3K4ME3_AND_H3_H3K27ME3 [1069] Genes with high-CpG-density promoters (HCP) bearing histone H3 dimethylation at K4 (H3K4me2) and trimethylation at K27 (H3K27me3) in brain. 56 1.58 e-6

SCHLOSSER_SERUM_RESPONSE_DN [712] Cluster 4: genes down-regulated in B493-6 cells (B lymphocytes) upon serum stimulation but not affected by MYC [GeneID=4609]. 42 1.9 e-6

BHAT_ESR1_TARGETS_NOT_VIA_AKT1_DN [88] Genes bound by ESR1 [GeneID=2099] and down-regulated by estradiol [PubChemID=5757] in MCF-7 cells (breast cancer). 12 4.16 e-6

#TOP TEN C2-CGP analysis

Gene Set Name [# Genes (K)] Description # Genes in Overlap (k) p value

TTGTTT_V$FOXO4_01 [2061] "Genes with promoter regions [-2kb,2kb] around transcription start site containing the motif TTGTTT which matches annotation for MLLT7: myeloid/lymphoid or mixed-lineage leukemia (trithorax homolog, Drosophila); translocated to, 7" 125 2.42 e-7

V$FOXO4_01 [243] "Genes with promoter regions [-2kb,2kb] around transcription start site containing the motif RWAAACAANNN which matches annotation for MLLT7: myeloid/lymphoid or mixed-lineage leukemia (trithorax homolog, Drosophila); translocated to, 7" 24 3.26 e-5

"AAAGACA,MIR-511 [202]" "Targets of MicroRNA AAAGACA,MIR-511" 20 1.41 e-4

"TAGCTTT,MIR-9 [236]" "Targets of MicroRNA TAGCTTT,MIR-9" 22 1.61 e-4

V$FOXJ2_02 [237] "Genes with promoter regions [-2kb,2kb] around transcription start site containing the motif AYMATAATATTTKN which matches annotation for FOXJ2: forkhead box J2" 22 1.71 e-4

TGTTTGY_V$HNF3_Q6 [738] "Genes with promoter regions [-2kb,2kb] around transcription start site containing the motif TGTTTGY which matches annotation for FOXA1: forkhead box A1" 49 2.25 e-4

AACTTT_UNKNOWN [1890] "Genes with promoter regions [-2kb,2kb] around transcription start site containing motif AACTTT. Motif does not match any known transcription factor" 103 2.94 e-4

V$HTF_01 [72] "Genes with promoter regions [-2kb,2kb] around transcription start site containing motif NNWWWWNGMCACGTCATYNYWNNN. Motif does not match any known transcription factor" 10 4.87 e-4

V$PXR_Q2 [256] "Genes with promoter regions [-2kb,2kb] around transcription start site containing the motif RRGGTYANTRNM which matches annotation for NR1H4: nuclear receptor subfamily 1, group H, member 4" 22 5.03 e-4

V$SRY_01 [224] "Genes with promoter regions [-2kb,2kb] around transcription start site containing the motif AAACWAM which matches annotation for SRY: sex determining region Y" 20 5.51 e-4

# TOP TEN C3 analysis


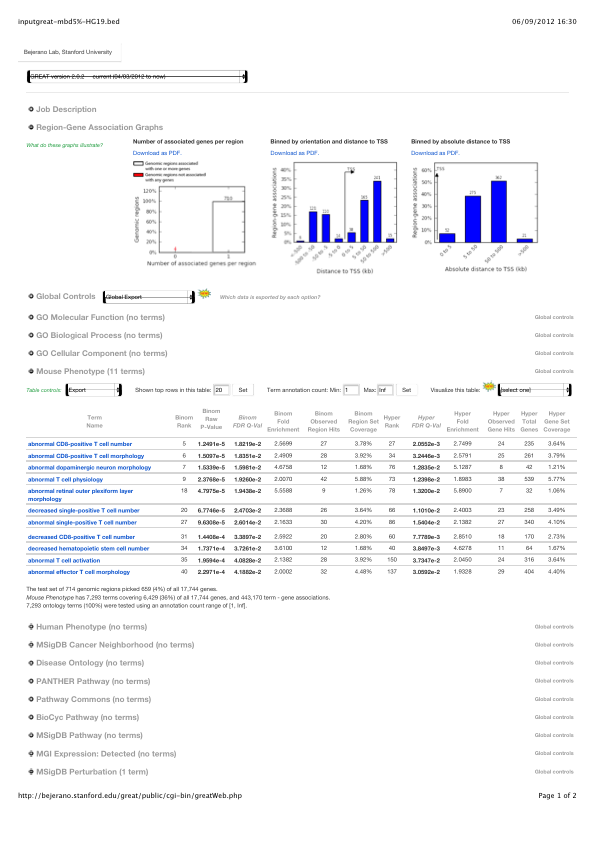


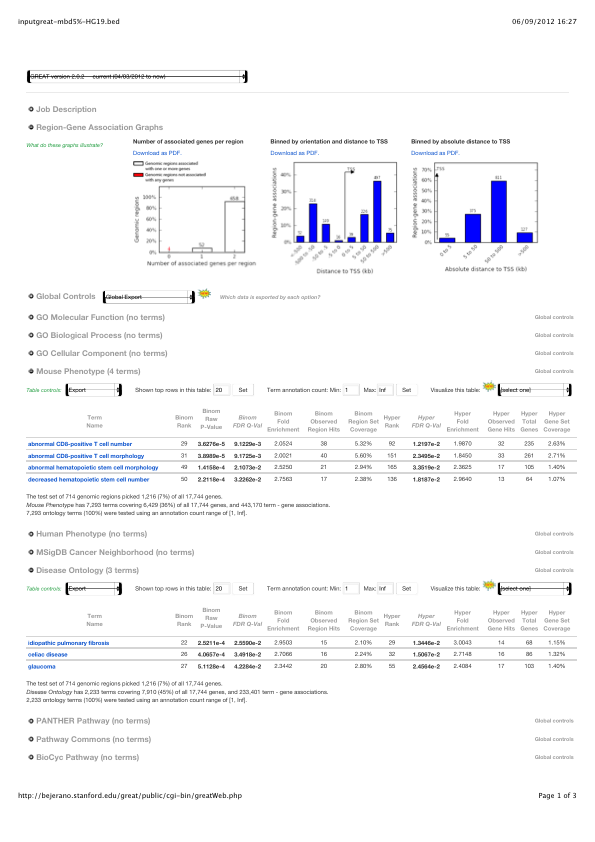


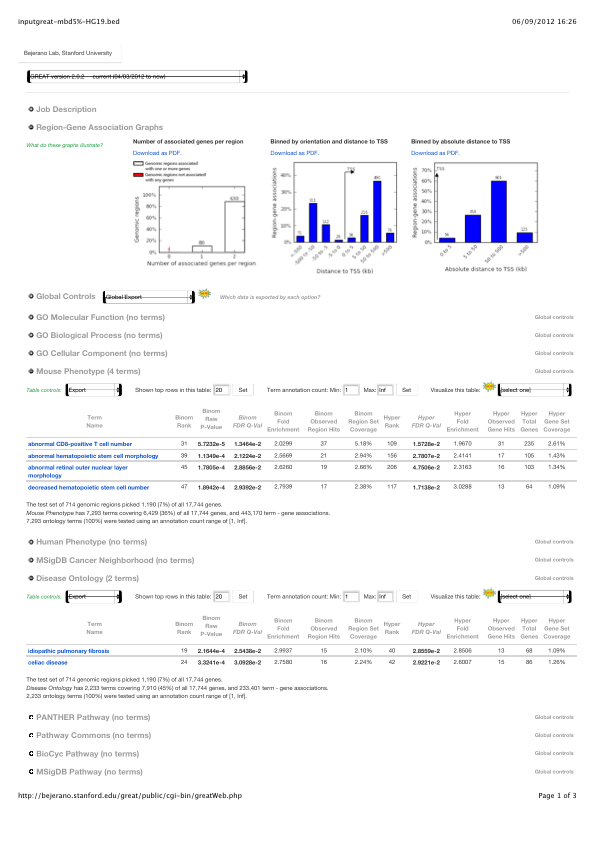

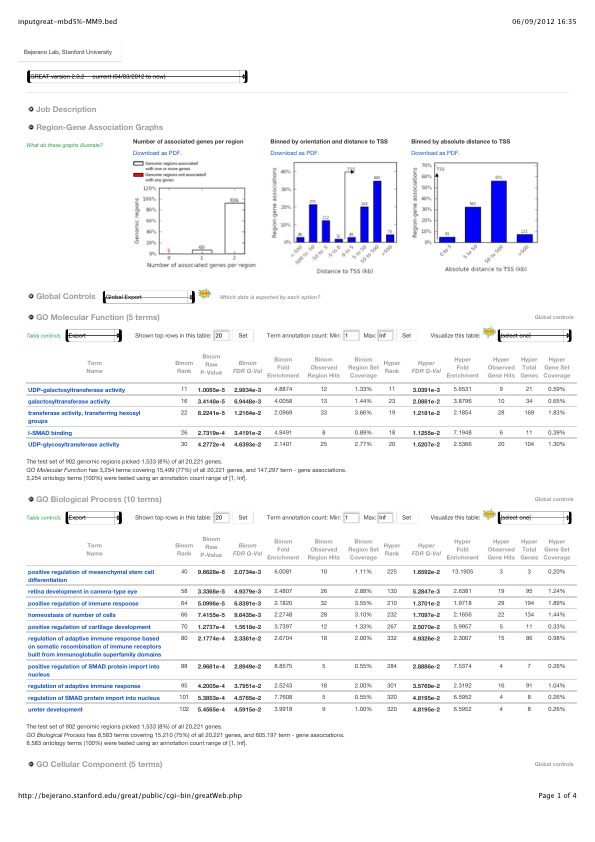

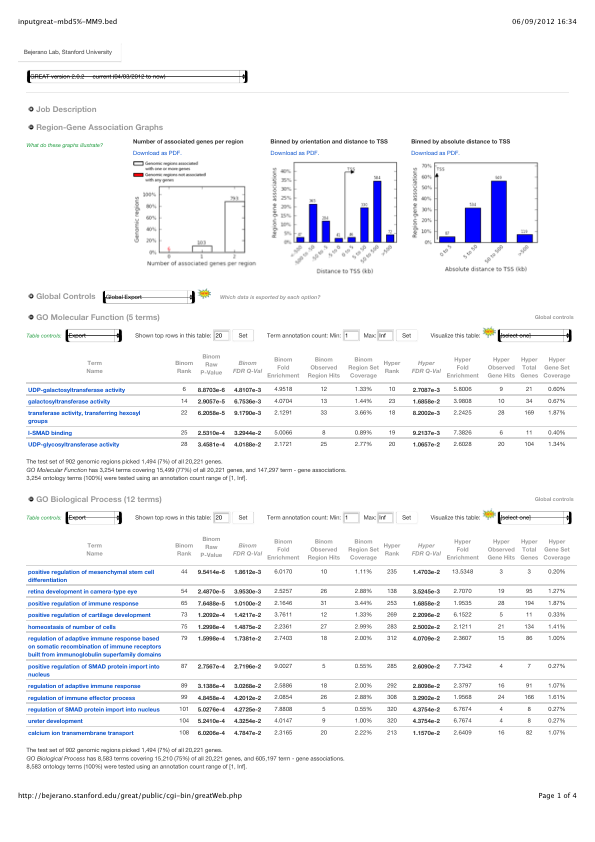

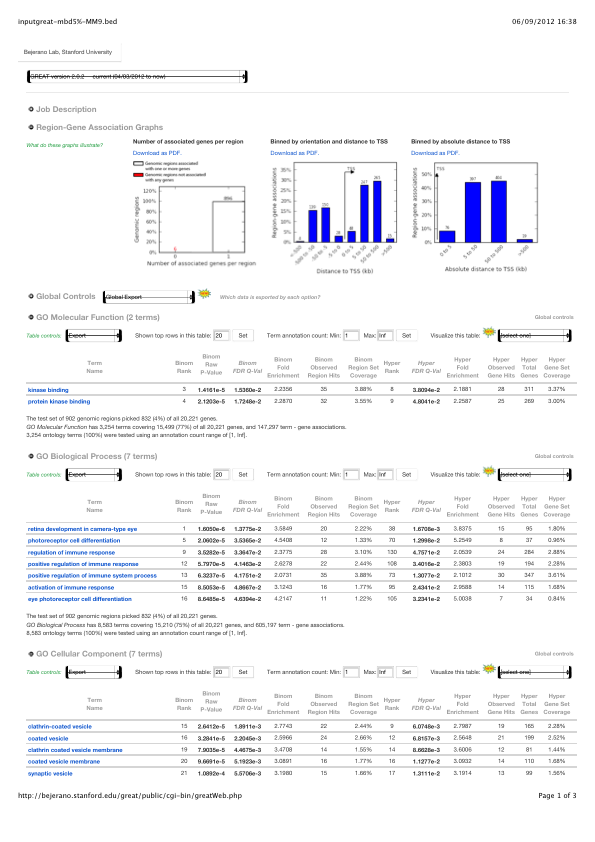

Supplement: Dataset S7 — DMR-neighbouring genes (MBD-Seq), Ontology, PvC. Ontology of the mapped differentially methylated regions-associated gene set (maternal low protein compared to control). File C2CGP: Overlap matrix by differentially expressed gene list (vertical) and gene sets (horizontal) that represent expression signatures of chemical and genetics perturbation (CGP) in MSigBD collection C2. File C3: Assuming an hypothetical differential expression for the DMR-neighbouring genes, overlap matrix by this gene list (vertical) and gene sets (horizontal) that contain genes that share a conserved cis-regulatory motif in promoters and 3-UTRs in MSigBD collection C3. File Summary and Description: Extensive description of the gene sets shown in Dataset S7. File outputgreat H19 basal+extension: Results from annotating human orthologs regions with GREAT rule basal+extension. File outputgreat H19 nearest: Results from annotating human orthologs regions with GREAT rule nearest gene. File outputgreat H19 two nearest: Results from annotating human orthologs regions with GREAT rule nearest two genes. File outputgreat MM9 basal+extension: Results from annotating mouse orthologs regions with GREAT rule basal+extension. File outputgreat MM9 nearest: Results from annotating mouse orthologs regions with GREAT rule nearest gene. File outputgreat MM9 two nearest: Results from annotating mouse orthologs regions with GREAT rule nearest two genes. (DOCX) [file pone.0082989.s007.docx]
