# Supplementary material for: Genome-Wide Methylation and Gene Expression Changes in Newborn Rats following Maternal Protein Restriction and Reversal by Folic Acid
Source: PLoS One. 2013 Dec 31;8(12):e82989. doi: 10.1371/journal.pone.0082989 (PMC3877003; doi:10.1371/journal.pone.0082989)
Supplement: Dataset S9 — Primers for GEX validations. The primers used for validation of differentially expressed gene probes in both maternal low protein compared to control and in maternal low protein compared maternal low protein supplemented with folic acid. (DOCX) [file pone.0082989.s009.docx]

Primers for Q-PCR

Geminin

F 5’TGAG**T**TGCCAAAAGGCTTGTCC 140bp

R 5’AGATCACATGCTTCCTGGGTG

MCM6

F 5’AAGA**G**TTCCAGGGTAGCG 160bp

R 5’GGGTAGACTCTGTAGAACTC

AURKB

F 5’TCAAAGACG**T**CTCAATCTGGC 145bp

R 5’CCTTGTTCTCAGTCAACTTCTG

Dnmt1

F 5’ GAA**C**GGAACACTCTCTCTCACTCAG 150bp

R 5’ TCACTGTCCGACTTGCTCCTC

Dnmt3b

F 5’ GTACACCAGAGACCAGAG 128bp

R 5’TCAGAGCCATCTCCATCATCC

Dnmt3a

F 5’ AGCAGTGACACCCCCAAGGAC 137bp

R 5’ GCTGGGCTCCCCTCCTCAGG

Glycin methyltransferase

F 5’CACAGGAGTGGACTCGATTATGC 135bp

R 5’ACCCACTTGTCAAAGGCTG

HAT1 126bp

F 5’ GGAGGCAGATGACGTTGAAGGCAA

R 5’ TGGAGGAGGGTTCCAAATGGCTTG
