# Supplementary material for: Genome-Wide Methylation and Gene Expression Changes in Newborn Rats following Maternal Protein Restriction and Reversal by Folic Acid
Source: PLoS One. 2013 Dec 31;8(12):e82989. doi: 10.1371/journal.pone.0082989 (PMC3877003; doi:10.1371/journal.pone.0082989)
Supplement: Dataset S10 — Primers for MBD validations. The primers used for validation of differentially methylated regions in maternal low protein compared to control, as illustrated in panel A of Figure 2 . (DOCX) [file pone.0082989.s010.docx]

| **Gene** | **Location of DMR** | **FDR,%** | **Fold enrichment** | **Genic** | **Primers** |
| --- | --- | --- | --- | --- | --- |
| **Agtrap** | Chr5: 165,156,757-165,157,718 | 5 | 7.88 | intronic | F_TTTGGTTTGTTTGTTTAGAAAG  R_CTAAAACCCTTCTCCTACTAC  Primers designed to forward strand |
| **Fgf5** | Chr14: 12,713,062-12,714,431 | 5.91 | 4.36 | downstream | F_AGTTTGTGTGGTAGGATTTGG  R_TACCCAACCTTTCAATCCATC  Primers designed to complement strand |
| **Dnmt1** | Chr8: 19,965,118-19,965,567 | 6.28 | 11.55 | intronic | F_1_ _ TAGAGGGTTATTTTAGTAATAGTTAT  F_2_ _ TAAAGGTAAGATTTAAAGAAAGG  R _ TATACATAATTTATCTTCCTCAAAC  Primers designed to forward strand |
| **MCM6** | Chr13: 41,055,046-41,056,193 | 12.44 | 4.6 | intronic | F_ TTTGGTAAATGGAGTTGGGATATAA  R_CTTTACAACATCTCTAAACAAATCTATCC  Primers designed to forward strand |
| **SFMBT^[[1]](#footnote-1)^** | Chr16: 6,176,260-6,178,343 | 0 | 21.31 | intronic | F_ AGTTTGTTGGTTGGATTAGATAG  R_CTAATATTAATTTATATCAACCTAACAAAATCC  Primers designed to complement strand |

Agtrap **validation intronic**

**Chr5:165156757-165157718; FDR 5, fold enrichment 7.88**

**PCR fragment 346 bp with 10 CpGs analysed**

**6 control livers, 27 clones (2 methylated)**

**5 livers, 25 clones (19 methylated)**

**Fisher’s exact test P <0.0012, calculations for clones**

**F_TTTGGTTTGTTTGTTTAGAAAG**

**R_CTAAAACCCTTCTCCTACTAC**

**Primers designed to forward strand**

Fgf5 **validation downstream**

**Chr14:12713062-12714431; FDR5.91, fold enrichment4.36**

**PCR fragment 278 bp with 13 CpG analysed**

**3 controls, 19 Clones**

**3 LP livers, 19 Clones**

**The two-tailed P value equals 0.0878**

**F_AGTTTGTGTGGTAGGATTTGG**

**R_TACCCAACCTTTCAATCCATC**

**Primers designed to complement strand**

Dnmt1 **validation Intronic**

**Chr8: 19,965,118-19,965,567; FDR6.28%, fold enrichment 11.55**

**PCR fragment 512 bp with 16 CpG analysed**

**6 Controls, 38 clones**

**5 LP, 33 clones**

**P value =0.0156 for whole region of 14 CpGs,**

**CpGs 15,16 outside of DMR**

**P value 0.0065 for CpG 3**

**0.0339 for CpG 4**

**F132 _ TAGAGGGTTATTTTAGTAATAGTTAT**

**F192 _ TAAAGGTAAGATTTAAAGAAAGG**

**R704 _ TATACATAATTTATCTTCCTCAAAC**

**Primers designed to forward strand**

MCM6 **validation intronic**

**Chr13: 41,055,046-41,056,193; FDR12.44%, fold enrichment 4.6**

**PCR fragment 350 bp with 5 CpG analysed**

**8 control livers, 53 clones (32 unmethylated CpGn1)**

**6 LP livers, 42 clones (11 unmethylated CpGn1)**

**p=0.0399 Fisher’s exact test for Cp****G1**

**F531 TTTGGTAAATGGAGTTGGGATATAA**

**R884 CTTTACAACATCTCTAAACAAATCTATCC**

**Primers designed to forward strand**

SFMBT **validation Intronic failed to validate**

**Chr16: 6176260-6178343; FDR 0, fold enrichment 21.31**

**5 control livers, 31 clones**

**7 LP livers, 32 clones**

**F_ AGTTTGTTGGTTGGATTAGATAG**

**R_CTAATATTAATTTATATCAACCTAACAAAATCC**

**Primers designed to complement strand. DMR is located in repeats. Reverse primer is designed in unique sequence, forward – in repeats**

**Expected products: 81,118,154,191, 227, 264, 301, 338 bp ...**

**Methylation percentages for validated regions**

|  | Animal group | Total CG sites | Methylated CG | % methylation |
| --- | --- | --- | --- | --- |
| Agtrap | C | 270 | 225 | 83 |
|  | LP | 250 | 240 | 96 |
| Fgf5 | C | 247 | 217 | 88 |
|  | LP | 247 | 241 | 98 |
| MCM6 | C | 265 | 205 | 77 |
|  | LP | 220 | 196 | 89 |
| Dnmt1 | C | 532 | 465 | 87 |
|  | LP | 642 | 428 | 93 |

1. This region is located in a stretch of repeats. Could not be validated [↑](#footnote-ref-1)
