# Supplementary material for: Genome-Wide Methylation and Gene Expression Changes in Newborn Rats following Maternal Protein Restriction and Reversal by Folic Acid
Source: PLoS One. 2013 Dec 31;8(12):e82989. doi: 10.1371/journal.pone.0082989 (PMC3877003; doi:10.1371/journal.pone.0082989)
Supplement: Table S1 — Stats of MBD regions, PvC. Descriptive statistics of hypermethylated regions, which are found in maternal low protein compared to control and the relevant numbers of Human and Mouse orthologous regions employed in ontological analysis. (DOCX) [file pone.0082989.s012.docx]

| **Genomic regions**  **MLP vs. C (no: 1183)** | **Length** | **Fold Enrichment** | **False discovery rate** | **E-value** | **No. of Tags** |
| --- | --- | --- | --- | --- | --- |
| **Mean** | 804.29 | 8.59 | 4.90 | 178.01 | 108.18 |
| **Median** | 729.5 | 7.79 | 4.94 | 126.75 | 75 |
| **Max** | 5062 | 117.59 | 5 | 3100 | 3617 |
| **Min** | 237 | 2.46 | 0 | 121.79 | 18 |
| **St Dev** | 384.61 | 5.83 | 0.42 | 315.62 | 148.74 |
| **Q. 0%** | 237.00 | 2.46 | 0.00 | 121.79 | 18 |
| **Q. 25%** | 572.75 | 6.36 | 4.93 | 123.94 | 44 |
| **Q. 50%** | 729.50 | 7.79 | 4.94 | 126.75 | 75 |
| **Q. 75%** | 952.00 | 9.60 | 4.97 | 129.36 | 128 |
| **Q. 100%** | 5062.0 | 117.59 | 5.00 | 3100 | 3617 |

Q. = quantile

St Dev = standard **deviation** (square root of variance)

E-value = -10log10(P-value)

**Repeats**

L1_Rn 70

dust 42

trf 29

L1_Rat1 16

B3 13

(other) 773

NA,s 240

**Stable ID (Ensemble ID)**

9 regions 3:1 (3 labels have been assigned 3 regions each)

122 regions 2:1 (61 labels have been assigned 2 regions each)

1052 regions 1:1 (1052 labels have been assigned to 1 region each)

1183 regions (number of hyper-methylated regions found in PvC FDR5% Liver, P1)

**Distance**

Min 7

1^st^ q. 14,400

Median 39,903

Mean 83,065

3^rd^ q. 96,948

Max 1,409,917

**Genomic Annotation**

**Intronic**: 474 regions

**Upstream**: 361 regions

**Downstream**: 320 regions

**Other** (5’/3’/UTR, exons): 28 regions

| UCSC LiftOver: MLP vs. C |  | species | # regions MBD-Seq FDR5% |
| --- | --- | --- | --- |
| Rat2Human/Mouse |  | RAT (RN4) | 1183 |
|  |  | MM9 | 901 |
|  |  | HG19 | 713 |
|  |  |  |  |
