# Supplementary material for: Genome-Wide Methylation and Gene Expression Changes in Newborn Rats following Maternal Protein Restriction and Reversal by Folic Acid
Source: PLoS One. 2013 Dec 31;8(12):e82989. doi: 10.1371/journal.pone.0082989 (PMC3877003; doi:10.1371/journal.pone.0082989)
Supplement: Table S2 — Diet-phenotype association. Description of the dam diets and of the pup phenotypes associated with dam diets. Folic acid supplementation (extra folate) = 4 mg/kg. Diets are matched for energy. No evidence of fetal programming due to high COH. (DOCX) [file pone.0082989.s013.docx]

| ANIMAL GROUP | DIET(mother)  (% to normal) | PHENOTYPE (pup) | INVESTIGATED |
| --- | --- | --- | --- |
| C | Standard- meets requirement | Normal blood pressure | yes |
| C+F | Standard rat diet + folic acid supplementation | No strong, consistent phenotype [Engeham et al 2010] | Not in this work |
| MLP | Low protein (50% less); higher carbs CHO (9% more), same fat content [Langley and Jackson 1994, Clin Science] | Hypertensive rat, vascular dysfunction  Low weight at birth. Nephron deficit. | yes |
| MLP+F | Same as LP + folic acid supplementation | Normal blood pressure and vessel function, almost normal weight at birth.  No nephron deficit | yes |

*Folic acid supplementation (extra folate) = 4mg/kg*

*Diets are matched for energy. NB: No evidence of fetal programming due to high COH.*
