# Supplementary material for: Genome-Wide Methylation and Gene Expression Changes in Newborn Rats following Maternal Protein Restriction and Reversal by Folic Acid
Source: PLoS One. 2013 Dec 31;8(12):e82989. doi: 10.1371/journal.pone.0082989 (PMC3877003; doi:10.1371/journal.pone.0082989)
Supplement: Table S3 — Number of total and unique reads per library. Number of total and unique reads per library. (DOCX) [file pone.0082989.s014.docx]

| MBD-Seq: MACS Quality Control |  |  |  |  |  |  |  |  |  |
| --- | --- | --- | --- | --- | --- | --- | --- | --- | --- |
|  | **Control** |  | **MLP** |  |  |  | **MLP+F** |  |  |
| No of tags | 16008758 |  | 32741244 |  |  |  | 23087218 |  |  |
| No. of unique tags | 13322026 |  | 26145395 |  |  |  | 17306868 |  |  |
| Duplication level^%^ | Below 15% |  | Below 15% |  |  |  | Below 15% |  |  |

^%^ MACS duplication index ranges between 0 (no duplication) and 1 (all clonal).

Rule of thumb: duplication should be kept below 20% (index = 0.20).
