# Supplementary figures and images for: Genome-Wide Methylation and Gene Expression Changes in Newborn Rats following Maternal Protein Restriction and Reversal by Folic Acid
Source: PLoS One. 2013 Dec 31;8(12):e82989. doi: 10.1371/journal.pone.0082989 (PMC3877003; doi:10.1371/journal.pone.0082989)

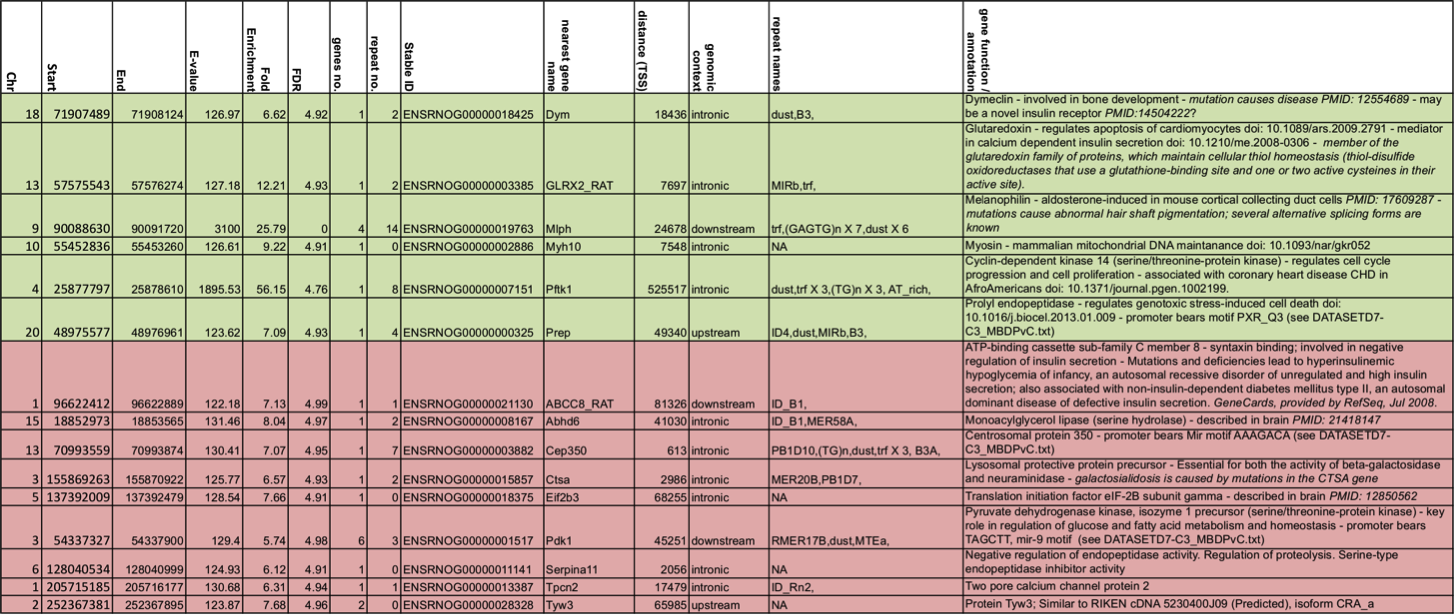

Supplement: Figure S1 — 15 DEX genes that bear DMRs. Genomic details and functional information for 15 differentially expressed genes bearing methylation marks (DMRs) in Maternal Low Protein compared to Control at P1 (FDR 5%). Up/Down-regulation is indicated by red and green highlights respectively. The overlap between the total number of mapped DMRs-neighboring genes and the total number of differentially expressed genes was not statistically significant (p = 0.69). (TIFF) [file pone.0082989.s015.tif]

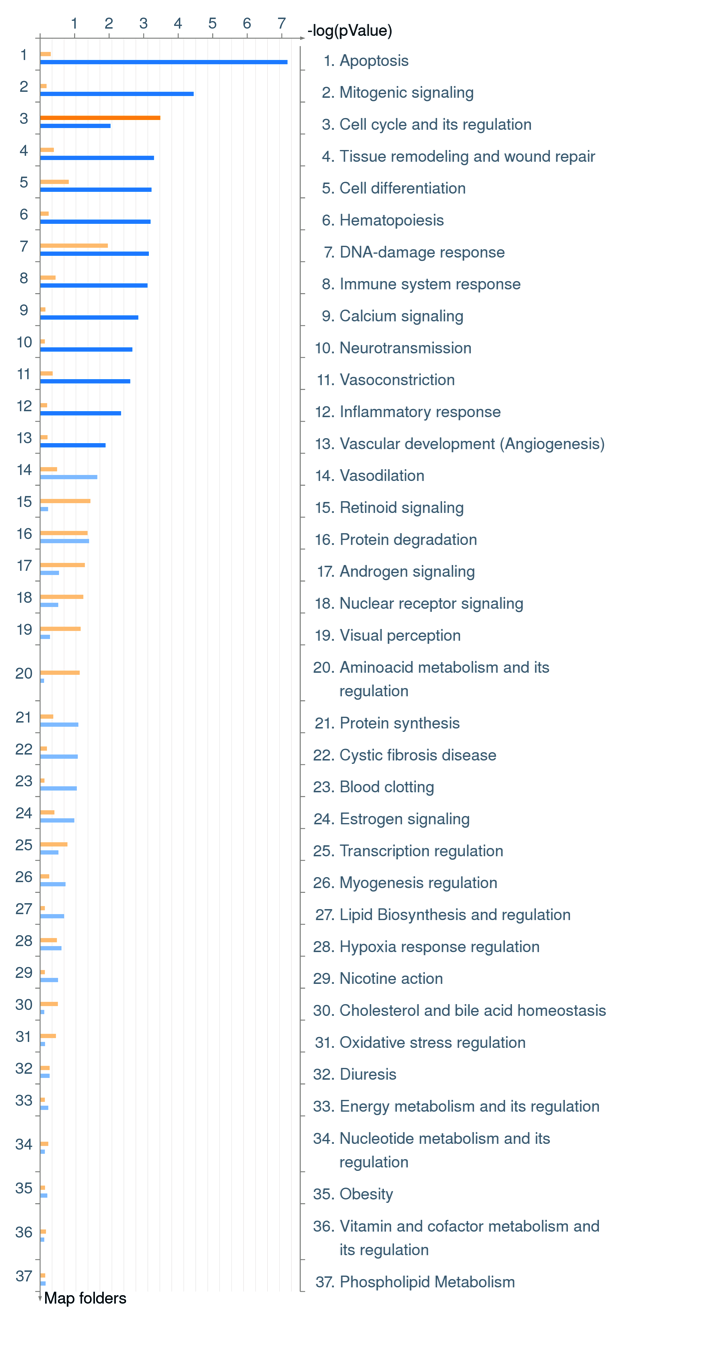

Supplement: Figure S2 — GENEGO picture, Word file with color keys. Comparative ontological analysis of DMR-neighboring and differentially expressed gene sets, which are respectively represented in blue and orange in the bar chart. (TIFF) [file pone.0082989.s016.tif]

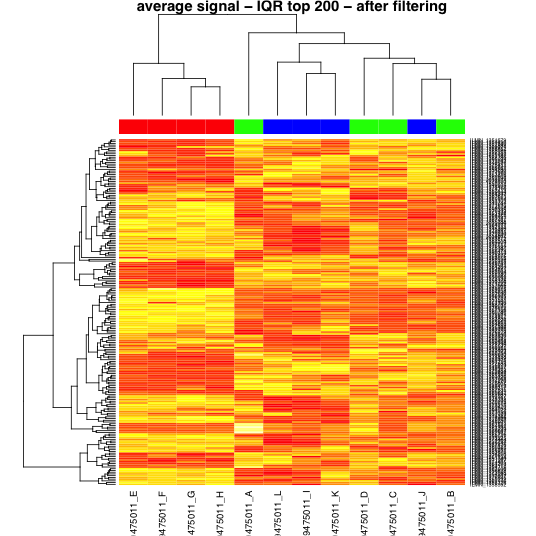

Supplement: Figure S3 — GEX quality control: heatmap, PvC. Heatmap of the top highly expressed gene probes, average signal after filtering low quality probes. Diet of control C represented by green bars, MLP+F group by blue bars, and MLP diet group by red bars. (TIFF) [file pone.0082989.s017.tif]

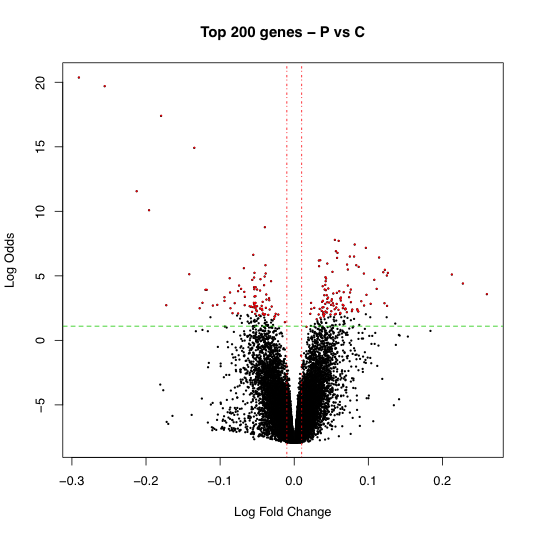

Supplement: Figure S4 — GEX quality control: volcano plot, PvC. Volcano plot of the differentially expressed gene probes in comparison to C of programmed group P = MLP. The top 200 highly significant gene probes are depicted with red dots. Vertical red lines correspond to fold changes equal to +1 and −1 (Log FC = +−0.01) and horizontal green lines corresponds to probability 0.75 (odds = 3, log-odds = 1.098612). (TIFF) [file pone.0082989.s018.tif]
